# Results of the joint IAEA/EEAE Intercomparison exercise on radioanalytical characterization of NORM samples in the European region

**DOI:** 10.1093/rpd/ncaf003

**Published:** 2025-02-20

**Authors:** Konstantinos Karfopoulos, Filipa Domingos, Govert de With, Bogusław Michalik, H Burçin Okyar, Christos Maramathas, Nikos Salpadimos, Constantinos Potiriadis, Valentina Neculae, Alžbeta Ďurecová, Veronika Drábová, Miroslav Pinak

**Affiliations:** Greek Atomic Energy Commission (EEAE), Environmental Radioactivity Monitoring Unit, 60092 P.C. 15341, Agia Paraskevi, Athens, Greece; Earth Sciences Department, Laboratory of Natural Radioactivity & Instituto D. Luiz, University of Coimbra, Faculty of Sciences and Technology, Rua Sílvio Lima, 3030-790, Coimbra, Portugal; Nuclear Research and Consultancy Group (NRG), Utrechtseweg 310, 9034, NL-6800 ES Arnhem, the Netherlands; Silesian Centre for Environmental Radioactivity (GIG PIB), Central Mining Institute – National Research Institute, Plac Gwarków, 1, Katowice 40-166, Poland; International Atomic Energy Agency (IAEA), Vienna International Centre, Wagramer Strasse 5, 1400 Vienna, Austria; teleDOS Laboratories S.M. P.C.—teleDOS Nuclear Tech, 102 Apostolou Paulou Str., 20131, Corinth, Greece; Greek Atomic Energy Commission (EEAE), Environmental Radioactivity Monitoring Unit, 60092 P.C. 15341, Agia Paraskevi, Athens, Greece; Greek Atomic Energy Commission (EEAE), Environmental Radioactivity Monitoring Unit, 60092 P.C. 15341, Agia Paraskevi, Athens, Greece; Institute for Nuclear Research—RATEN ICN-Pitesti, 1 Street Campului, Mioveni 115400, Romania; Regional Public Health Authority Banská Bystrica, Cesta k nemocnici 1, 975 56, Banská Bystrica, Slovak Republic; Public Health Authority of the Slovak Republic, Ružinovská 8, 820 09, Bratislava, Slovak Republic; International Atomic Energy Agency (IAEA), Vienna International Centre, Wagramer Strasse 5, 1400 Vienna, Austria

**Keywords:** Naturally Occurring Radioactive Material, Natural Radionuclides, Intercomparison Exercise, Activity Concentration Index *I*, Phosphate Ore, Phosphogypsum

## Abstract

Naturally Occurring Radioactive Material (NORM) may pose radiological risks across various industrial processes. Characterizing NORM is challenging due to radionuclide diversity, complex material matrices, and the multiple analytical techniques required. This study documents an Intercomparison Exercise (ICE) on the radioanalytical characterization of NORM, organized by International Atomic Energy Agency and EEAE to evaluate participants’ abilities to implement appropriate radioanalytical techniques and promote harmonization, thus contributing to ongoing optimization efforts into radiation protection of workers and the public. Thirty-one laboratories from 21 countries participated and determined the activity concentrations of long-lived radionuclides such as ^40^K, ^238^U, ^226^Ra, and ^232^Th in two ICE items, most through gamma spectrometry. Improper handling and insufficient testing of equilibria status within uranium and thorium series were key sources of unsatisfactory results. Notably, laboratories’ accreditation status did not correlate with analytical accuracy. Overall, study findings highlight improvements are needed in sample preparation, assumptions’ validation and measurement uncertainty budget estimation procedures.

## Introduction

### Motivation

Numerous industrial processes involving Naturally Occurring Radioactive Material (NORM) follow an extensive life cycle that includes the extraction of materials, manufacture of products and by-products, generation of discharges, residues, and wastes, and finally, the decommissioning of the facilities [1]. In these industries, NORM might not be the prevailing hazard. However, NORM should be under routine radiological assessment, as the radiation dose may exceed dose constraints.

NORM is defined as a radioactive material containing no significant amounts of radionuclides other than Naturally Occurring Radionuclides (NORs) [2]. The potential radiation exposure from NORM in numerous industrial sectors has been widely recognized since the 1980s. Recent recommendations have emphasized the need for fostering inclusive and sustainable capacities to monitor, record, and control occupational exposure at workplaces. Furthermore, materials that are of concern due to the presence of NORs are generally placed under regulatory control when the activity concentrations exceed exemption levels. For the primordial radionuclides ^238^U, ^232^Th and their progenies the generic exemption level is 1 kBq·kg^−1^ [3]. For a generic exemption decision, in case of disequilibrium, the radionuclide with the highest activity concentration in the decay series should be taken into account. For ^40^K a generic exemption level of 10 kBq·kg^−1^ applies [3]. These exemption levels are applicable to bulk material, and any material with an activity concentration below this level is deemed safe, as the annual dose for either workers or public is expected to be <1 mSv.

For NORM or, more generally, NOR-containing materials that are used as a building material in dwellings, a reference level of 1 mSv per year for the occupant applies to the excess dose received from external exposure to gamma radiation [4]. This excludes any additional committed dose from ^222^Rn or ^220^Rn exhalated from building materials into indoor air. To assess compliance with the reference level the activity concentration index *I* is used as calculated by the following formula:


(1) \begin{equation*} I=\frac{C_{Ra-226}}{300}+\frac{C_{Th-232}}{200}+\frac{C_{K-40}}{3000} \end{equation*}

The index is based on the activity concentrations (Bq·kg^−1^) of the radionuclides ^226^Ra, ^232^Th and ^40^K, and an index value of <1 (*I* < 1) or 6 (*I* < 6) when a material will be used as bulk or superficial building material respectively, ensures that the reference level will not be exceeded.

To enable correct classification of all these NOR-containing materials and to perform a robust dose assessment when classified, accurate determination of the NORs is of key importance. Only under these conditions conformity to the regulatory requirements can be adequately checked, and dose assessment as well as optimization can be performed to minimize external and internal exposures using appropriate protection measures. This paper will report the findings from a Regional Intercomparison Exercise (ICE) that was carried out among radioanalytical laboratories in the European region of the International Atomic Energy Agency (IAEA). The study includes an extensive scientific evaluation of the laboratories’ performance for NORM analysis and makes an inventory on the current measurement practice in the region. Through this ICE, valuable insights in the laboratory practices will be provided, contributing to the refinement of measurement techniques, the enhancement of accuracy in radiological assessments, and the development of robust protocols for the characterization of NORM.

### Radiological characteristics of NORM

**Fig. 1:**
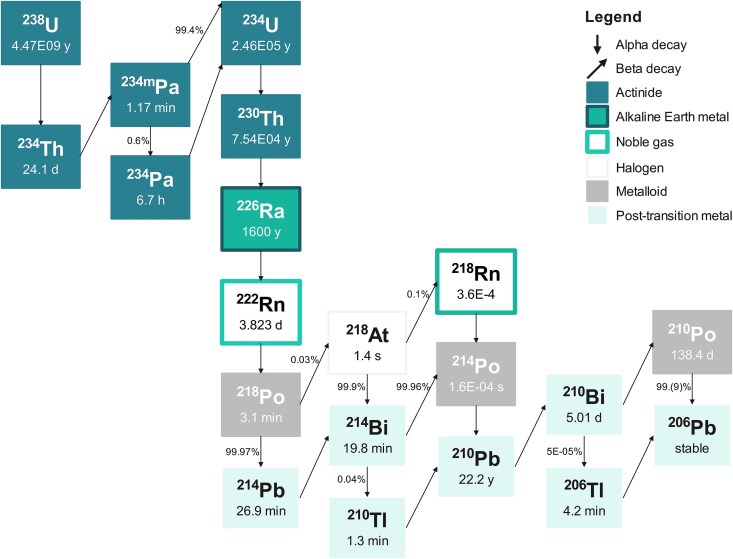
Uranium (^238^U) decay series (10 elements). Nuclear data were retrieved from LNHB [7].

**Fig. 2:**
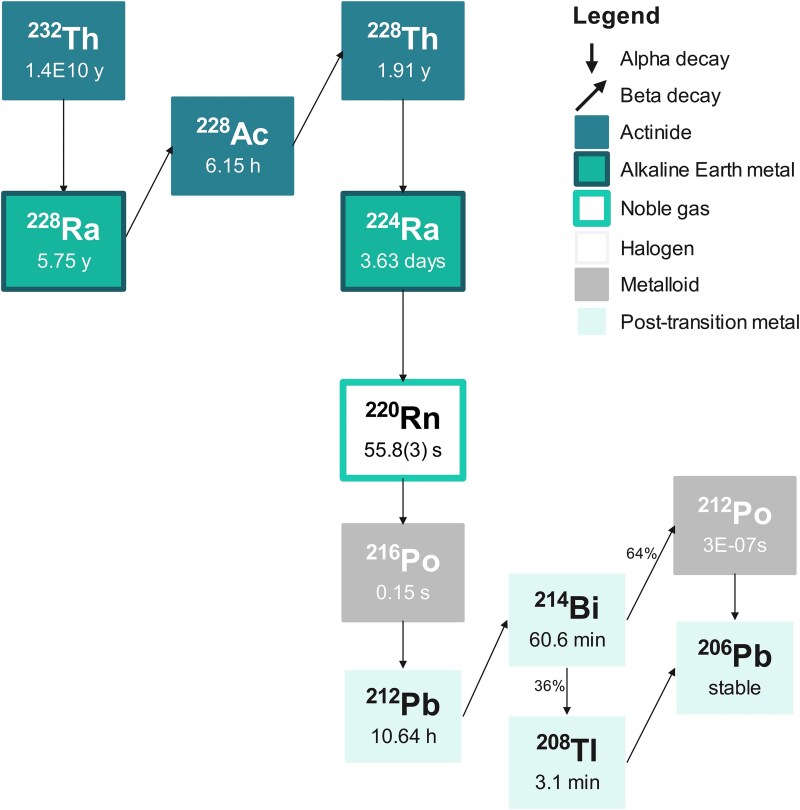
Thorium (^232^Th) decay series (eight elements). Nuclear data were retrieved from LNHB [7].

The radionuclides of interest are the primordial radionuclides ^238^U, ^235^U and ^232^Th together with their progenies and the single radionuclide ^40^K. Uranium-238 (Fig. 1) and ^232^Th (Fig. 2) are heading decay series with multiple progeny nuclides each. The radionuclides constituting each decay series of ^238^U, ^235^U and ^232^Th will be in equilibrium when located in an isolated system for an adequately long time [5, 6]. In such case the activity concentration of all members of the series can be determined at once, by estimating the activity concentration of one member of the series, typically the one that can be easily and reliably determined.

However, with the exception of some geological samples and unprocessed raw materials where the influence of external factors is negligible, an equilibrium between all the members of each decay series is frequently absent. Such disruptions are caused by natural and/or anthropogenic processes and -depending on the technology employed, the prevailing environmental conditions and the different chemical properties between the series’ elements—some radionuclides will be totally or partially removed from the series while others remain in place. Only a few members of the decay series have a half-life that is long enough therefore they can be affected by external conditions that disturb the equilibrium for a considerable period of time. This disruption leads to variations in activity concentrations and the formation of subseries that are shown in Table 1.

**Table 1:** Parent radionuclides and their respective progeny(ies).

**Parent nuclide**	**Progeny**
^238^U	^234^Th, ^234m^Pa, ^234^Pa
^226^Ra	^222^Rn, ^218^Po, ^214^Pb, ^214^Bi, ^214^Po, ^210^Pb, ^210^Bi, ^210^Po
^222^Rn	^218^Po, ^214^Pb, ^214^Bi, ^214^Po
^210^Pb	^210^Bi, ^210^Po
^232^Th	^228^Ra, ^228^Ac
^228^Ra	^228^Ac
^228^Th	^224^Ra, ^220^Rn, ^216^Po, ^212^Pb, ^212^Bi, ^208^Tl (36%), ^212^Po (64%)
^220^Rn	^216^Po
^212^Pb	^212^Bi, ^208^Tl (36%), ^212^Po (64%).

The presence of such disequilibrium and the formation of the subseries has fundamental implications for the measurement of the NORs and, eventually, the assessment of the radiation exposure. For example, determination of the individual subseries is a key step, and provisions must be put in place to adequately determine the radionuclides heading such subseries. Therefore, determination of the radionuclidic composition should not only involve analysis of a sample using radiometric techniques but also consider the sample’s source history to make a preliminary identification of the processes that may impact equilibria in the various subseries. Additionally, it should involve the quantification of radionuclides that are not directly measured, by taking due consideration that the state of equilibrium in the measured sample may well differ from the original sampled material, since its composition may be altered by sampling, sample treatment, and/or the decays in the isolated aliquot(s) through time. Finally, information regarding radionuclide activity ratios can aid in refining measurement uncertainty or even enable the indirect measurement of a specific radionuclide. All these aspects are considered within this study and extensively discussed.

### IAEA regional ICE

The IAEA is committed to promote an internationally harmonized approach for the safe management of NORM and maintains international safety standards and guidelines to optimize radiation exposure at the workplace by taking into account the graded approach. It also offers technical assistance to the Member States in applying these safety standards and guidelines in practice. With the support of IAEA’s Regional Technical Cooperation Project RER/9/155, a Regional ICE on Radioanalytical Characterization of NORM Samples was jointly organized by the IAEA and the Greek Atomic Energy Commission (EEAE). The aim of this exercise is to assess the capabilities of the Member States in the region to apply radioanalytical techniques (i.e. gamma spectrometry, alpha spectrometry and liquid scintillation counting [LSC]) for the characterization of NORM in line with the IAEA Safety Standards.

Under this ICE, NORM samples were prepared by the Environmental Radioactivity Monitoring Unit of EEAE. The ICE was conducted between July and November 2022 with the final full participation of 31 radioanalytical laboratories, 29 of which were officially designated and two more were added at a later time, from 21 countries within the European region of the IAEA. Each participant was provided two types of material: a phosphate ore (PO) and a phosphogypsum (PG) sample. The participants were requested to determine the activity concentrations of the NORs by applying radioanalytical techniques of their own choice. Individual results were evaluated in terms of trueness and compatibility [8], on the basis of the z-scores and ζ-scores respectively, in accordance with ISO [9]. Furthermore, special attention was given to the laboratories’ practices that influence analytical performance, such as the handling and procedures for assessment of the state of equilibria, sample preparation, sealing techniques, measurement geometry setups, calibration methods performed, and corrections applied.

The most common method implemented for NORM analysis was high-resolution gamma-ray spectrometry (HRGS) [10]. This technique allows the determination of almost all gamma-emitting radionuclides present in a sample. However, to date there are no standardized procedures for the evaluation of NOR in NORM based on the above radiometric technique, except the more generic procedures described in ISO 18589-3 [11]. A European Standard CEN/TS 17216:2023 on the application of HRGS for radiological analysis of building materials is currently under development. This standard is specifically developed for the determination of ^226^Ra, ^232^Th and ^40^K in construction products. Other radiometric techniques that are used for NORM analysis include alpha spectrometry and LSC after radiochemical separations. While these methods are generally not widely applied for routine measurement of NORM samples, they are well recognized. Therefore, they were included in this ICE to allow for a comprehensive intercomparison.

## Materials and methods

### Sample materials

The production of phosphate fertilizer is the primary activity of the phosphate industry accounting for the vast majority of global phosphate production. Phosphate deposits contain calcium and phosphorus, mainly as tricalcium phosphate Ca_3_ (PO_4_)_2_ [12, 13], as well as NOR at a trace or ultra-trace level. The main radionuclides contributing to external gamma radiation exposure are ^214^Pb and ^214^Bi from the ^238^U decay series, as well as ^228^Ac, ^212^Pb and ^208^Tl from the ^232^Th decay series. Overall, the activity concentrations in currently exploited POs range from 0.1 to 3 Bq/g for radionuclides in the ^238^U decay series and from 0.1 to 0.4 Bq/g for radionuclides in the ^232^Th decay series [13].

Phosphogypsum is generated as a by-product of the phosphoric acid-based fertilizer industry, consisting of a CaSO_4_·2H_2_O matrix [14]. During the production process, the different radionuclides released from the raw material follow either the phosphoric acid or the generated by-product, according to their respective elemental chemical behaviors [15]. Uranium present in the phosphate rock solubilizes and goes into the phosphoric acid fraction whereas radium is co-precipitated with the PG, which is usually stored in land surface areas close to the processing plants. Phosphogypsum properties, in terms of density, strength, compressibility and permeability, depend on the source of the phosphate rock, the reaction conditions in the attack tank, and on the deposition, age, location, and depth of the PG within the stack. As such, the moisture content can range from 8% to 30%, depending on the properties of the material [13]. The ^238^U activity concentrations are mostly below 0.1 Bq/g, however, higher values may occur due to lower recovery levels of phosphate in the attack tank. The activity concentrations of ^226^Ra and its progenies are expected to range from 0.2 to 3 Bq/g, for material derived from sedimentary PO, and to be lower than 0.7 Bq/g for material derived from igneous ore [13, 16]. It is also evident that activity concentrations vary between stacks and between different levels in stacks, depending on factors such as age and source rock characteristics [13].

### Sample preparation

A joint ICE was organized within IAEA’s technical cooperation project RER/9/155 focusing on the determination of radionuclides in NORM samples aiming to improve measurement capabilities in the region [17]. Within the context of the ICE, ~20 kg of raw material of PO and PG were collected from a phosphoric acid production unit in Greece. The materials were homogenized, and subsequently, a sieve shaker was used to separate the particles with diameter lower than 2 millimeters. The moisture content of each material was assessed by drying a representative portion of the samples and measuring their weight before and after oven drying. The final dry weight was measured after at least 15 hours of oven drying at 80°C. The moisture was calculated as 1.30 ± 0.01% for PO and 28.70 ± 0.28% for the PG sample. Subsequently, the bulk materials were homogenized again and subdivided into 40 samples each.

The methodology for homogeneity testing followed ISO [9]. The procedure was performed at EEAE by measuring two portions of each sample using a low background gamma-spectrometer consisting of a Broad Energy Germanium (BEGe) detector with a relative efficiency of ~50%, a Digital Signal Analyzer (DSA1000) and gamma-spectrometry software installed in an interconnected computer. For both materials, the chosen measurand for the homogeneity check was the radioactivity of the natural gamma-emitting radionuclides in each aliquot, expressed as gammas counted by the gamma spectrometry system. Amongst all the gamma lines recorded in each spectrum and the possible combinations of their net areas, the sum of the three net area counts of 186, 609, and 1764 keV peaks was utilized for the homogeneity assessment. This combination was chosen on purpose, aiming to reduce the uncertainty of the results, due to its relatively better counting statistics (higher sum values). The measurements were performed under identical high repeatability conditions and lasted 3 hours each. According to the between-sample standard deviations (*s_s_*), which were determined through the spectrometric analyses’ results, the PO samples were deemed homogenous within 7.5%, while the PG samples were considered as homogenous within 15%. The observed inhomogeneities were reasonable for well-prepared ICE test items consisting of NORM. Moreover, in this ICE, since it was decided beforehand the inhomogeneities to be included in the standard deviation for proficiency assessment (*σ_PT_*) for each radionuclide and material (see §2.4), the criterion *s_s_* ≤ 0.3·*σ_PT_* for acceptability of homogeneity could be relaxed [9].

Finally, after assuring the sufficient homogeneity of the test items, samples were prepared and packed by the EEAE under the auspices of IAEA (Fig. S1). Each sample was given a specific code and then distributed to participants, during the Regional Workshop on Radioanalytical Characterization of NORM samples, which was held in Athens in July 2022 (IAEA ME-RER9155–2201679). A total of 80 samples were originally distributed amongst 35 laboratories from 25 countries. Of these, only 31 laboratories from 21 countries reported their results. The list of the officially designated participants who participated in this ICE is presented as supplementary material (Appendix S1, Table S1). Laboratories were informed of their individual performance during the Regional Workshop on Evaluation and Finalization of Intercomparison Exercise on Radioanalytical Characterization of NORM Samples, held in Ankara in July 2023 (IAEA ME-RER9155–2301382).

### Methodology

Laboratories were requested to analyze both samples and determine the activity concentrations of ^40^K and radionuclides from the ^238^U, ^235^U and ^232^Th decay series, expressed in Bq/kg dry mass. The estimated activity concentration value for each identified radionuclide had to be reported accompanied by its combined standard uncertainty and the respective minimum detectable activity concentration (MDA), to form a metrologically complete result [18]. Laboratories were also asked to provide details on the uncertainty budget components. The radionuclides under reporting were the participants’ free choice and were not limited to the long-lived parent ones. Furthermore, no requirements on the sample preparation, measurement protocol or type of radiation detection were imposed. Laboratories were free to use their own routine protocols and procedures, and any of the facilities or equipment they had at their disposal.

Along with the samples, a questionnaire was distributed to all participating laboratories to obtain relevant information about their methodology. Details were asked on the sample preparation, such as sample drying/processing, details of the container, including its sealing and waiting time prior to gamma measurement. For the sample’s measurement, details on the applied detection technology, the calibration, and any relevant corrections (e.g. true coincidence summing and self-absorption) were asked, as well as on counting time and data analysis. Information about the application of standardized methods, the available quality management system (QMS) and their ISO accreditation status were also requested. After receiving the samples, the participants had four months to report their results.

### Performance evaluation

Laboratories’ performance evaluation was based on the comparison of the results with a consensus value estimated from participants’ results. The consensus or assigned value (${x}_{PT}$) for each radionuclide corresponded to the robust average (x^*^) that was calculated according to Algorithm A in ISO [9]. This algorithm consists of an iterative computational procedure that excludes outliers and is terminated when no changes occur in the third significant figure of the robust mean (x^*^) and robust standard deviation (s^*^), simultaneously [9]. To optimize the assigned values and their uncertainties, some participant results were excluded from the dataset used as input in Algorithm A, based on three criteria of reliability. Firstly, results with a measurand’s value below two times the MDA were excluded since they could be substantially inaccurate, due to the fact that below this level, the results are expected to suffer from a standard uncertainty higher than 20%, approximately, when probabilities of the error of the first and second kind are α = β = 0.05, as is usually the case in ionizing radiation metrology [10, 18, 19]. Secondly, metrologically incomplete results (e.g. not containing the MDA values) were excluded, since they could not be checked against the first criterion. In fact, the absence of the MDAs’ assessment and reporting could be an indication of an insufficient QMS in the respective laboratory, a limited participant’s experience in the method(s) applied, or of the use of a newly introduced or unvalidated analytical procedure, all of which could compromise analytical quality and negatively impact the corresponding results. Lastly, given the high moisture content of the PG sample, the activity concentrations -expressed on dry mass basis—reported by participants with unacceptable performance in moisture determination (z score ≥ 3), were expected to be strongly biased. Hence, these results, as well as the results of the laboratories that did not assess or report moisture, were excluded from the computation of the assigned values for the concentration of radionuclides in PG. The range of measured moisture content of the PO sample was very narrow, averaging 1%, which demonstrates that the material stability was commendable during the course of the ICE. Thus, the impact of moisture on the computation of the assigned values was considered negligible even when the results of a few participants who did not report or apply corrections for dry mass were included. It is also worth noting there were no unacceptable performance among the laboratories which provided an estimation of moisture. Ultimately, <35% of the results were excluded upon the application of the three reliability criteria listed above. The validation of this optimization procedure is presented as Supplementary Material (Appendix S2 and Table S2; [20]).

After optimization, using the output of Algorithm A and considering *p* as the number of results used to compute *x_PT_* and s^*^, the uncertainty of the assigned values, $u\left({x}_{PT}\right)$, was calculated according to the following equation:


(2) \begin{equation*} u\left({x}_{PT}\right)=1.25\cdot \frac{s^{\ast }}{\sqrt{p}} \end{equation*}

According to ISO [9], the standard deviation for proficiency assessment (${\sigma}_{PT}$) should be limited when the robust standard deviation is either very small or very large. These limits must ensure that an acceptable performance score is obtained when measurement error is fit for the most challenging intended use while also ensuring that the results unfit for purpose receive an action signal. In this ICE, the following limiting criteria were applied for ${\sigma}_{PT}$:


(3) \begin{equation*} {\sigma}_{PT}=\left\{\begin{array}{c}{s}^{\ast },\mathrm{when}\ u\left({x}_{PT}\right)<{s}^{\ast }<{x}_{PT}\cdot \sqrt{u{(a)}^2+u{(h)}^2+u{(i)}^2}\ \\{}\\{}{x}_{PT}\cdot \sqrt{u{(a)}^2+u{(h)}^2+u{(i)}^2},\mathrm{when}\ u\left({x}_{PT}\right)<{x}_{PT}\cdot \sqrt{u{(a)}^2+u{(h)}^2+u{(i)}^2}<{s}^{\ast}\\{}\\{}u\left({x}_{PT}\right),\mathrm{when}\ {x}_{PT}\cdot \sqrt{u{(a)}^2+u{(h)}^2+u{(i)}^2}<u\left({x}_{PT}\right)<{s}^{\ast}\end{array}\ \right. \end{equation*}

Here *u(a)* is the maximum relative standard uncertainty expected from radioanalytical techniques, *u(h)* is the relative uncertainty related to the heterogeneity of the moisture content among samples during the course of the ICE, and *u(i)* is the uncertainty component due to inhomogeneity of the samples (7.5% for PO and 15% for PG). A *u(a)* value of 20% was established for both samples based on two empirical facts. Firstly, the variability of the normally distributed results from a group of highly competent radioanalytical laboratories is usually smaller than 20%. Secondly, radiometric methods’ relative standard combined uncertainties and biases are almost always lower than 20% when (i) measurement duration is long enough; (ii) the equipment used is appropriately calibrated; (iii) the measurand’s value is higher than two times the MDA; and, in general, (iv) all the analytical procedures are well performed fully following a thoroughly evaluated laboratory protocol. A *u(h)* equal to 0.5% for PO and 10% for PG was calculated. The wide range of moisture values reported during the ICE especially for the latter material, suggests that either moisture was not stable during the ICE, or the procedures for estimating this parameter were not consistent among the participants. After combining the above uncertainty components in quadrature, the limiting factor in equation (3) became 21% for PO and 27% for PG.

Table 2 shows the assigned values (*x_PT_*), their uncertainty, s^*^ and ${\sigma}_{PT}$ for the PO and PG. In general, the estimated assigned activity concentrations were higher for PO, whereas the relative standard uncertainty was higher for PG. The absence of a statistically significant difference (at the 95% confidence level) between the activity concentrations of the ^238^U decay series’ radionuclides indicates secular equilibrium was established in PO. On the contrary, equilibrium was disturbed in PG, given the assigned values for ^238^U and ^234^Th were significantly lower than the values for their progenies (^226^Ra, ^214^Pb, ^214^Bi, and ^210^Pb). Established secular equilibrium in the ^232^Th decay series was also evident in the PO confirming the finding from ^238^U series, whereas in PG, the assigned value for ^232^Th tended to be lower than the assigned values for its progenies (^228^Ra, ^228^Ac, and ^228^Th) but not statistically different. The lower assigned value for ^232^Th stemmed from two reported activity concentration values of 5.6 Bq/kg which resulted from the application of the much more specific alpha spectrometry technique. These two results further suggest disequilibrium within the ^232^Th decay series in PG, as is the case within ^238^U series. Given the high probability of disequilibrium and the lack of information regarding the production date of PG, participants should only have reported results for ^232^Th and ^238^U if they were obtained by alpha spectrometry techniques. However, only two out of 13 participants reported alpha spectrometry results for ^232^Th and one out of 11 for ^238^U. Thus, the assigned values for ^232^Th and ^238^U in PG likely include a common bias and may deviate significantly from the true activity concentrations. Nevertheless, ${\sigma}_{PT}$ and $u\left({x}_{PT}\right)$ were found to be high enough and they may cover the potential common bias of those two assigned values.

**Table 2:** Assigned values as the robust average (x_PT_ = x^*^), assigned value uncertainty, *u(x_PT_)*, robust standard deviation (s^*^), and the standard deviation for proficiency testing (${\sigma}_{PT}$) for the PO and PG samples in Bq/kg (dry mass).

Analyte of interest (NOR)	Phosphate ore sample (results in Bq/kg dry mass)				Phosphogypsum sample (results in Bq/kg dry mass)			
	*x_PT_*	*u(x_PT_)*	s^*^	${\sigma}_{PT}$	*x_PT_*	*u(x_PT_)*	s^*^	${\sigma}_{PT}$
^40^K	30.7	2.1	7.3	6.6	15.1	2.3	5.5	4.1
^235^U	28.8	1.3	4.1	4.1	3.09	0.89	2.0	0.89
^238^U	550	53	146	118	47	10	19	12
^234^Th	570	59	133	122	33.4	2.5	4.8	4.8
^226^Ra	539	24	93	93	348	14	47	47
^214^Pb	522	24	58	58	335	23	53	53
^214^Bi	524	25	60	60	339	23	53	53
^210^Pb	446	56	180	95	316	16	46	46
^232^Th	43.6	2.4	6.5	6.5	24.8	9.0	19	9.0
^228^Ra	47.7	2.1	4.4	4.4	36.4	3.5	7.4	7.4
^228^Ac	45.8	1.2	3.1	3.1	32.9	2.1	4.7	4.7
^228^Th	52.9	4.3	9.1	9.1	30.8	5.7	12	8.3
^212^Pb	47.3	1.4	3.5	3.5	19.26	0.74	1.7	1.7
^208^Tl	20.1	2.0	5.1	4.3	8.6	1.1	2.7	2.3

Laboratories’ performance was evaluated through z scores (Equation 4) for trueness and zeta ($\zeta$) scores (Equation 5) for compatibility:


(4) \begin{equation*} {z}_i=\frac{x_i-{x}_{PT}}{\sigma_{PT}} \end{equation*}

(5) \begin{equation*} {\zeta}_i=\frac{x_i-{x}_{PT}}{\sqrt{u^2\left({x}_i\right)+{u}^2\left({x}_{PT}\right)}} \end{equation*}

Where ${x}_i$ and $u\left({x}_i\right)$ are the participant’s reported result and (combined) standard uncertainty, respectively. According to ISO [9], when the uncertainty of the assigned values is higher than $0.3\cdot{\sigma}_{PT}$ it cannot be deemed negligible and the use of z´ scores instead of z scores is recommended:


(6) \begin{equation*} {z}_i^{\prime }=\frac{x_i-{x}_{PT}}{\sqrt{{\sigma_{PT}}^2+{u}^2\left({x}_{PT}\right)}} \end{equation*}

Absolute results of these metrics lower than or equal to 2 were considered acceptable (A). Results higher than 2 but lower than 3 were given a warning signal (W) and results higher than or equal to 3 were marked as unacceptable (N). Repeated warnings (W) for a participant signal the need for further investigation towards possible optimizations in the method(s) applied and/or equipment setup, etc., while unacceptable results point out that extended corrective actions are required.

An evaluation of participants’ performance regarding precision was also attempted using the results of those metrics and considering the criteria described below. On one hand, a combined uncertainty *u(x_i_)* is consistent with an observed deviation *x_i_ – x_PT_* and could be considered correctly defined when | *ζ_i_* | < 3. On the other hand, an absolute *ζ_i_* below 3 could stem from a severely overestimated *u(x_i_)*, i.e. when *u(x_i_)* ≥ 1.5·s^*^ or in its more generic form *u(x_i_)/x_i_* ≥ 1.5·s^*^/x_PT_. For *u(x_i_)* to be neither overestimated nor underestimated severely, the conditions: | *ζ_i_* | < 3 and *u(x_i_)/x_i_* < 1.5·s^*^/x_PT_ should be fulfilled simultaneously. The precision can be deemed acceptable (A) when one of the following cases is true:

- | *ζ_i_* | ≤ 2 when *u(x_i_)/x_i_* ≤ *σ_PT_/x_PT_*;
- | *ζ_i_* | ≤ 2 when *σ_PT_/x_PT_* < *u(x_i_)/x_i_* < 1.5·s^*^/x_PT_ and *x_i_* < 2·MDA, or
- 2 < | *ζ_i_* | < 3 when *σ_PT_/x_PT_* < *u(x_i_)/x_i_* < 1.5·s^*^/x_PT_ and *x_i_* < 2·MDA.

A warning signal (W) is given for precision in the following situations:

- if 2 < | *ζ_i_* | < 3 when *x_i_* ≥ 2·MDA and *σ_PT_/x_PT_* < *u(x_i_)/x_i_* < 1.5·s^*^/x_PT_;
- when *u(x_i_)* is slightly underestimated or overestimated, i.e. if either 2 < | *ζ_i_* | < 3 when *u(x_i_)/x_i_* ≤ *σ_PT_/x_PT_*, or | *ζ_i_* | ≤ 2 when *x_i_* ≥ 2·MDA and *σ_PT_/x_PT_* < *u(x_i_)/x_i_* < 1.5·s^*^/x_PT_, respectively.

Finally, having the evaluation outcomes regarding trueness and precision of a reported result, a conclusion about its accuracy can be drawn [21]. Accuracy can be deemed acceptable (A) safely when trueness is acceptable, and precision is rated as “A” or “W”. A warning signal (W) for accuracy is generated when trueness is acceptable, but precision is unacceptable (N), or when trueness received a “W”, irrespective of precision score. Accuracy is deemed unacceptable (N) when trueness is unacceptable, irrespective of the precision score.

## Results

Two samples (PO and PG) were distributed to the participants. Overall, 31 laboratories reported results for ^40^K, ^235^U, members of the ^238^U decay series (^238^U, ^234^Th, ^226^Ra, ^214^Pb, ^214^Bi and ^210^Pb) and of the ^232^Th decay series (^232^Th, ^228^Ra, ^228^Ac, ^228^Th, ^212^Pb, and ^208^Tl). Most were determined through HRGS. Three laboratories reported results obtained applying alpha spectrometry directly for ^235^U, ^238^U, ^232^Th, ^228^Th and ^226^Ra and one laboratory reported a ^226^Ra result determined by LSC. The full results and a summary of the methodologies and procedures are presented as supplementary material (Appendix S3, Table S3). An overall assessment of the results is presented in section 3.1. and a more detailed assessment in section 3.2.

### Overall assessment

**Fig. 3:**
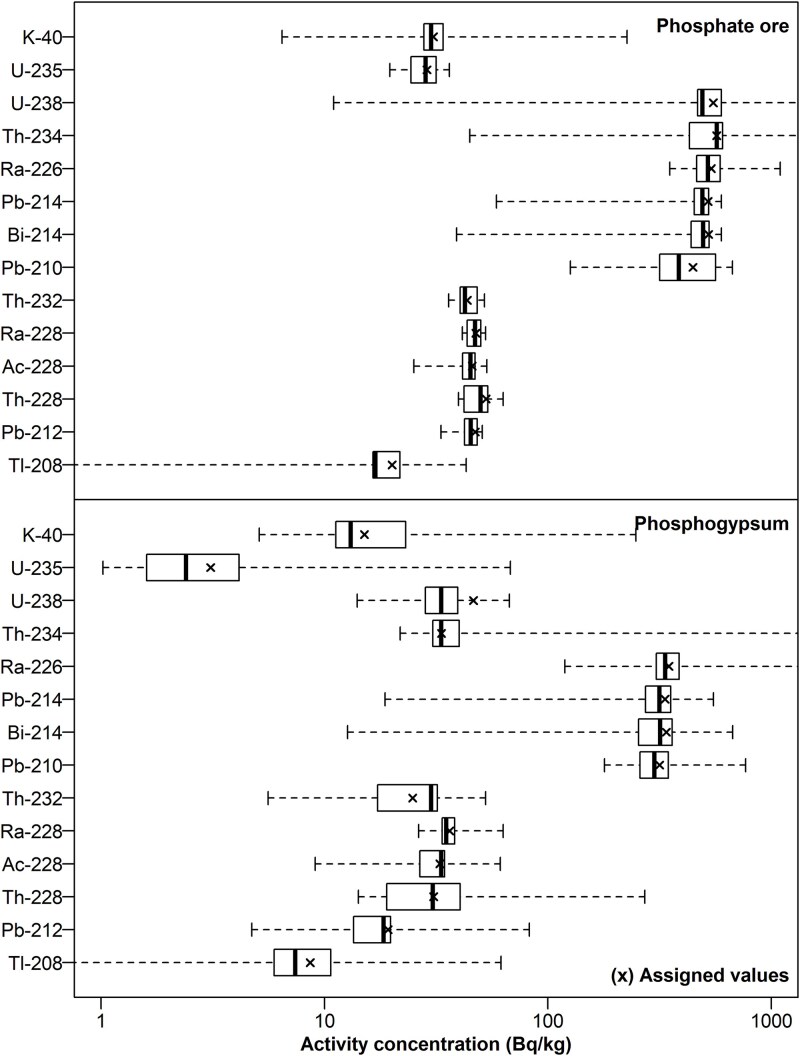
Box-and-whiskers plots of massic activity concentration of NOR (in Bq/kg) by material. the assigned values for each radionuclide are indicated by an x cross.

The variability of the activity concentration of NOR is illustrated in Fig. 3. Activity concentrations of all radionuclides are higher in PO compared to PG. This is particularly evident for ^235^U, ^238^U and ^234^Th, which differ in activity concentration by one order of magnitude between the samples. In PO, the members of the decay series of ^238^U present a similar distribution, with median, first and third quartiles values consistent with each other. The same conclusion can be drawn for members of the ^232^Th decay series apart from ^208^Tl. In PG, median ^238^U and ^234^Th activity concentrations are significantly lower than the activity concentration of their progenies. Hence, equilibrium in the ^238^U decay series cannot be assumed as established. The median activity concentrations are also less consistent between members of the ^232^Th decay series when compared to PO. The median ^208^Tl activity concentration averages 37% of the median activity concentration of its parents for PO, and 27% for PG, resembling the decay branching ratio of ^212^Bi to ^208^Tl (see Fig. 2). The medians are generally close to the assigned values (Fig. 3).

A summary of the performance evaluations of laboratories is presented in Tables 3 and 4. For PO, the percentage of accepted reported results ranged from 73% to 100% for trueness evaluations (z´ or z), from 52% to 89% for compatibility evaluations (*ζ*), and from 38% to 78% for precision (Table 3). For PG, the percentage of accepted results ranged from 53% to 92% for trueness, from 52% to 91% for compatibility, and from 27% to 91% for precision (Table 4). The percentage of accepted results by radionuclide in PO was almost always equal to or higher than the percentage of accepted results for the same analyte in PG, implying that laboratories had more difficulties assessing radionuclide concentrations in PG. Nonetheless, a good overall performance of laboratories was observed, given the percentage of warning and action signals is generally below 40% and the fact that, totally, more than 70% of the results were accurate (Tables 4 and 5, Figs. S2 and S3).

**Table 3:** Summary of the performance evaluations of the laboratories’ results for the PO sample. A – Acceptable; W – Warning signal; N – Not acceptable.

Analyte	Non-reporting labs (%)	Phosphate ore sample – Normalized evaluation scores											
		Trueness			Compatibility			Precision			Accuracy		
		A	W	N	A	W	N	A	W	N	A	W	N
^40^K	6.5	83	3	14	72	10	17	62	17	21	79	7	14
^235^U	48	94	6	0	89	6	6	78	17	6	94	6	0
^238^U	55	87	0	13	67	20	13	67	20	13	80	7	13
^234^Th	68	80	0	20	60	10	30	60	10	30	70	10	20
^226^Ra	10	83	3	14	62	3	34	62	3	34	66	21	14
^214^Pb	52	87	0	13	73	7	20	73	7	20	80	7	13
^214^Bi	52	87	0	13	60	13	27	53	20	27	73	13	13
^210^Pb	35	81	19	0	52	29	19	38	43	19	81	19	0
^232^Th	58	100	0	0	86	7	7	71	21	7	93	7	0
^228^Ra	74	100	0	0	88	13	0	75	13	13	88	13	0
^228^Ac	52	80	13	7	73	7	20	47	13	40	60	33	7
^228^Th	71	100	0	0	78	22	0	67	33	0	100	0	0
^212^Pb	52	80	13	7	67	20	13	40	27	33	60	33	7
^208^Tl	52	73	7	20	67	7	27	60	7	33	67	13	20
Participant group		Phosphate ore sample – Distribution of evaluation scores											
Accredited labs		87	7	6	67	13	20	54	20	26	73	20	6
Not accredited labs		84	2	14	74	9	16	71	13	16	82	4	14
Entire population		86	5	9	70	12	18	60	18	22	77	14	9

**Table 4:** Summary of the performance evaluations of the laboratories’ results for the PG sample. A – Acceptable; W – Warning signal; N – Not acceptable.

Analyte	Non-reporting labs (%)	Phosphogypsum sample – Normalized evaluation scores											
		Trueness			Compatibility			Precision			Accuracy		
		A	W	N	A	W	N	A	W	N	A	W	N
^40^K	26	74	9	17	70	13	17	57	22	22	74	9	17
^235^U	61	86	0	14	86	7	7	71	21	7	86	0	14
^238^U	65	91	9	0	91	9	0	91	0	9	91	9	0
^234^Th	71	78	11	11	78	0	22	67	0	33	67	22	11
^226^Ra	10	76	7	17	52	21	28	48	24	28	72	10	17
^214^Pb	52	73	7	20	67	7	27	67	7	27	67	13	20
^214^Bi	52	73	7	20	67	0	33	67	0	33	67	13	20
^210^Pb	35	81	14	5	62	0	38	52	5	43	57	38	5
^232^Th	61	92	8	0	77	15	8	77	15	8	92	8	0
^228^Ra	74	88	0	13	75	13	13	75	13	13	88	0	13
^228^Ac	52	87	0	13	67	13	20	47	13	40	60	27	13
^228^Th	68	90	0	10	70	20	10	70	20	10	90	0	10
^212^Pb	52	53	7	40	60	0	40	27	20	53	47	13	40
^208^Tl	52	67	20	13	60	13	27	47	13	40	60	27	13
Participant group		Phosphogypsum sample – Distribution of evaluation scores											
Accredited labs		79	11	10	64	13	23	55	14	31	69	21	10
Not accredited labs		76	1	22	75	4	21	64	13	22	75	3	22
Entire population		78	8	15	68	10	23	59	14	28	71	15	15

Higher acceptance rates and lower unreported rates in both samples were observed for ^40^K and NOR from the ^238^U decay series, which suggest laboratories had less difficulty determining these radionuclides compared to the members of ^232^Th series, whose concentration was relatively low (close to the levels commonly found in soils). Moreover, the scores of accepted precision evaluation results were almost always worse than the accepted z´ or z scores, indicating the laboratories had more difficulty assessing the combined uncertainty of their results than to determine the measurand values with sufficient trueness. The above are also supported by the overall distribution of evaluation scores by participant group for both samples (Tables 3 and 4).

The comparison of accredited and unaccredited laboratories shows that the performances regarding trueness of their results are equivalent (Tables 3 and 4). On the other hand, the results of the non-accredited laboratories were found substantially better in terms of precision compared to the results of the accredited ones, since for the PO 71% of the non-accredited laboratories’ results are of acceptable precision whereas the performance of the accredited ones is only 54%, and the respective scores for PG are 64% for non-accredited laboratories compared to 55% for accredited ones.

### Detailed intercomparison exercise results

#### Moisture

**Fig. 4:**
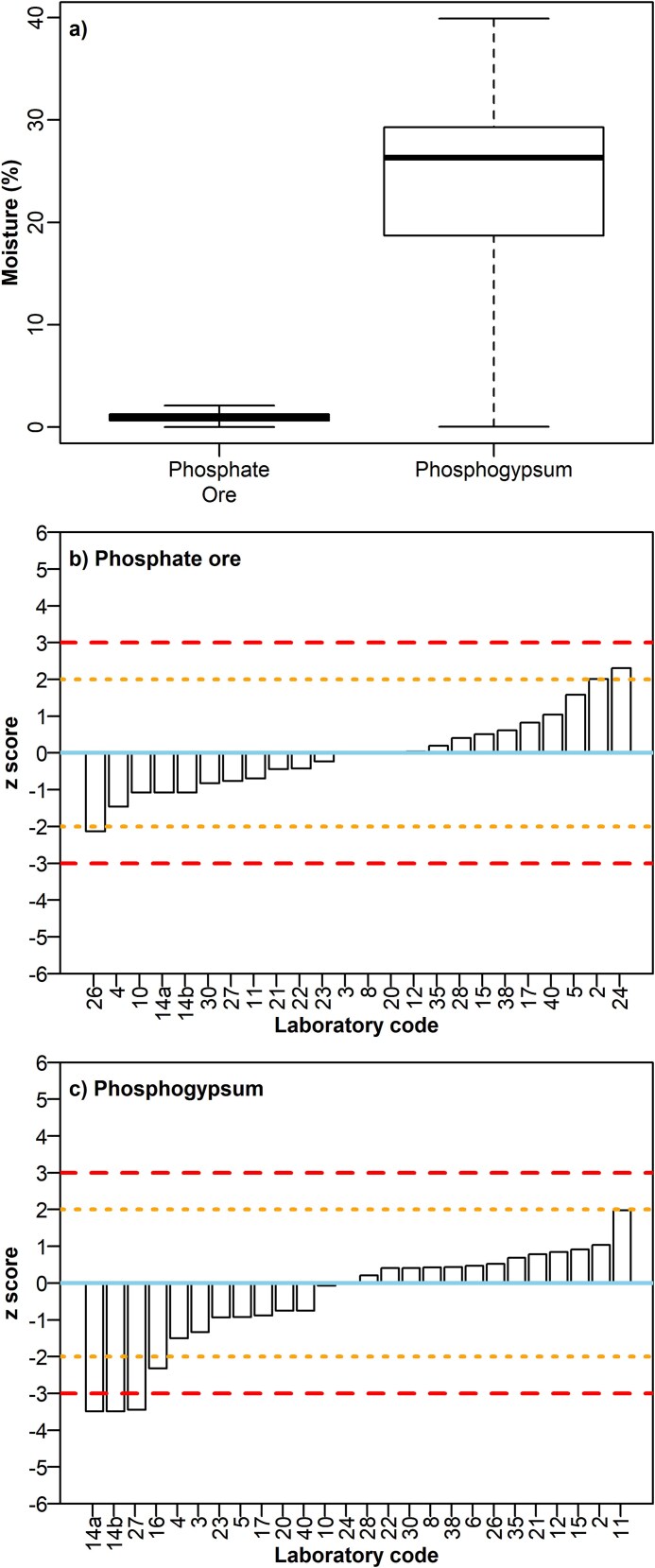
(**a**) Boxplot of moisture content values (%content on a wet mass basis) reported by participating laboratories per sample. Z scores of participating laboratories regarding determination of moisture in (**b**) PO and (**c**) PG. the continuous line represents an ideal score. The dotted lines represent the limits beyond which a warning is signaled. The dashed lines represent the limits beyond which the results are deemed unacceptable.

The reported moisture content (%content on a wet mass basis) ranged from 0% to 2.1% for PO, with a mean of 1% and assigned value 1.01 ± 0.12%. The moisture reported for PG ranged from 0.1% to 39.9% with a mean of 22% and assigned value 25.5 ± 1.9%, which agrees with the values recorded in literature [13]. Nearly all laboratories, except seven participants (namely #6, #9, #13, #16, #19, #29, and #34) who did not report moisture, had an acceptable performance regarding PO (Fig. 4). For PG, five laboratories (namely #9, #13, #19, #29, and #34) did not report the moisture content; from the reporting ones three had unacceptable results (#14a, #14b, and #27), while the others performed generally well.

#### Potassium-40

**Fig. 5:**
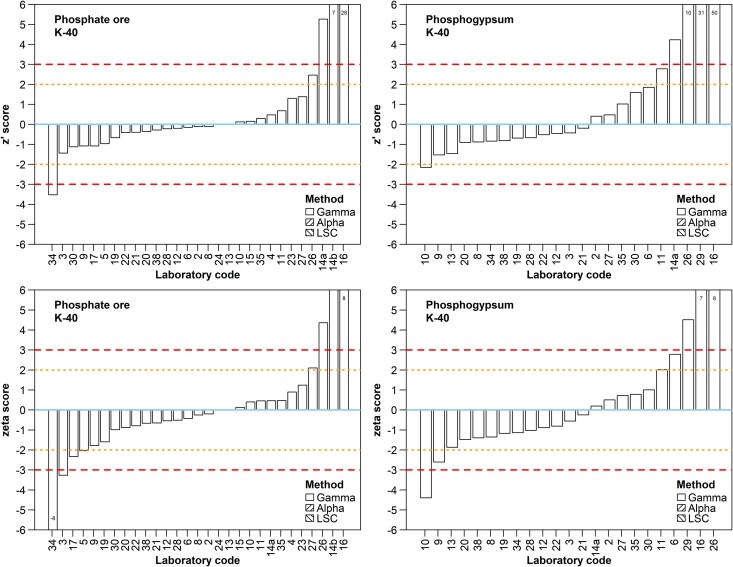
Z´ and ζ scores for ^40^K by participant for the PO and PG samples. Refer to the caption of Fig. 4 for a description of the line patterns used in z´ and ζ score plots.

Twenty-nine ^40^K results were reported for PO and 23 for PG. The reported activity concentration ranged from 6 to 226 Bq/kg for PO with a mean of 39 Bq/kg, and from 5 to 248 Bq/kg for PG, with a mean of 34 Bq/kg, whilst the assigned activities are 30.7 ± 2.1 and 15.1 ± 2.3 Bq/kg respectively. The lower number of reported results for PG as well as the slightly worse performance of the reporting laboratories can be attributed to the lower activity concentration of ^40^K in PG compared to PO (Fig. 5). In fact, some participants were not evaluated, since they correctly reported the result as below their detection limit or, equivalently, reported only the detection limit, which was comparable to the assigned value. Trueness, compatibility, and precision of 18 out of the 23 laboratories received an “A” or “W” score for PG, and of 23 out of the 29 laboratories for PO, advocating for their ability to properly assess ^40^K activity concentration and its uncertainty with easiness. Two laboratories (namely #14a and #16) grossly overestimated ^40^K activity concentration and at the same time did not estimate its uncertainty correctly in both materials, while four more (#14b, #26, #29 and #34) reported inaccurate ^40^K results for only one of the samples.

#### Uranium-235

**Fig. 6:**
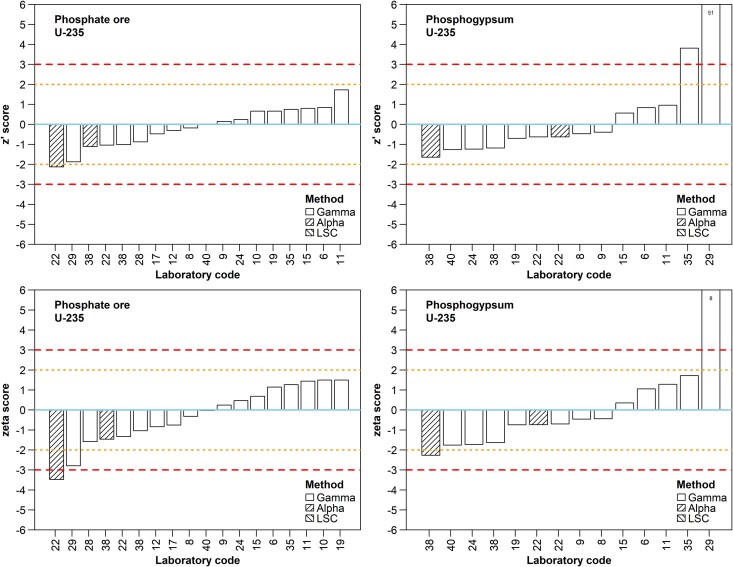
Z´ and ζ scores for ^235^U by participant for the PO and PG samples. Refer to the caption of Fig. 4 for a description of the line patterns used in z´ and ζ score plots.

Eighteen results were reported for PO and 14 for PG. The reported activity concentration ranged from 20 to 36 Bq/kg for PO, with a mean of 28 Bq/kg (assigned activity 28.8 ± 1.3 Bq/kg), and from 1 to 68 Bq/kg for PG, with a mean of 8 Bq/kg. The lower activity concentration of ^235^U in the PG material justifies both the lower number of results reported for this sample and the worse performance of the reporting laboratories, as the assigned value (3.09 ± 0.89 Bq/kg) is comparable to the MDA values reported by the participants. Most of the results which were determined through alpha spectrometry were found equivalent and close to the majority of gamma spectrometry results as well as to the assigned values (Fig. 6). The trueness score of all reporting laboratories except one (#22 which received a warning signal) was acceptable for the PO sample, whereas two laboratories (#29 and #35) provided unacceptable results for the PG sample against 12 ones which received an “A”. Similarly to ^40^K, precision scores were generally worse than the corresponding trueness performances for both materials. Finally, apart from three laboratories, the remaining reporting participants met the expected performance for ^235^U, determining this radionuclide with satisfactory accuracy.

#### Members of the Uranium-238 decay series

Apart from ^226^Ra, the z´ and ζ score plots for the members of ^238^U decay series are attached as supplementary material (Figs. S4 to S8). Laboratories reported ^238^U activity concentration ranging from 11 to 10 850 Bq/kg for PO and from 14 to 67 Bq/kg for the PG. Considering that the assigned value of ^238^U for PO is 550 Bq/kg, one laboratory (namely #34) reported a result 20 times higher than the “target” value. The same laboratory also overestimated severely the activity concentration of ^234^Th and ^226^Ra in both samples. This was probably due to reporting in time-integrated counts or in a unit system other than the widely adopted International System of Units (SI), despite instructions to report the activity concentrations in Bq/kg. Accuracy (trueness and precision) was acceptable for 22 out of 26 ^238^U results reported for both materials. Two laboratories (namely #9 and #10) received a warning signal, and another one (#16) provided highly underestimated measurand and uncertainty values for one of the samples.

The reported activity concentrations for ^234^Th ranged from 45 to 612 Bq/kg for PO and from 22 to 41 Bq/kg for PG (excluding the abnormal results from laboratory #34). For both samples, three results from two laboratories (namely #6 and #34) had unacceptable trueness and precision. A third laboratory (#13) had one acceptable and one warning score for trueness, but the corresponding reported uncertainty was significantly underestimated for both materials, leading to one warning signal and one unacceptable ζ or precision score. Two more laboratories provided results of acceptable trueness, but one of them (#5) underestimated the uncertainty and the other (#22) overestimated it at an unacceptable level, for only one of the samples.

**Fig. 7:**
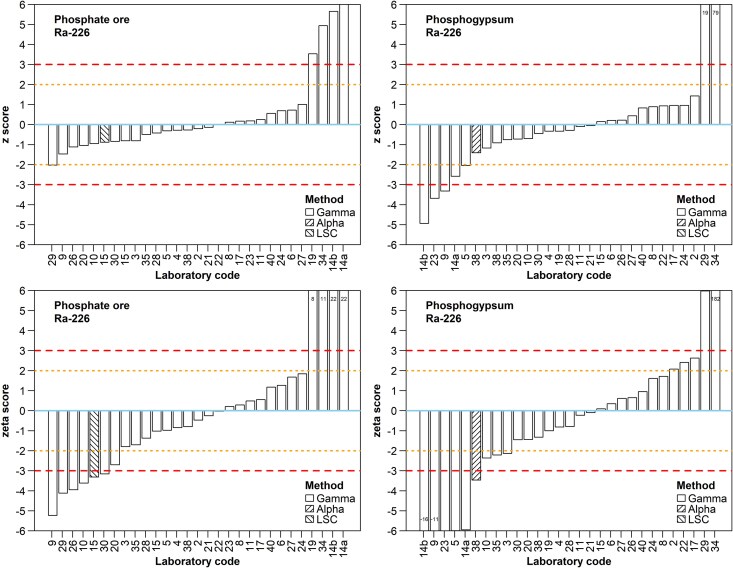
Z and ζ scores for ^226^Ra by participant for the PO and PG samples. Refer to the caption of Fig. 4 for a description of the line patterns used in z and ζ score plots.

Twenty-nine results were reported for ^226^Ra in each sample (Fig. 7). The reported activity concentrations ranged from 351 to 1096 Bq/kg for PO and from 119 to 1245 Bq/kg for PG (excluding laboratory #34). Four laboratories (namely #14a, #14b, #19, and #34) overestimated ^226^Ra activity concentration in PO by a factor of ~2, which may suggest an oversight of the interference of ^226^Ra and ^235^U 186 keV gamma-lines, leading the laboratories to attribute the measured signal entirely to ^226^Ra activity. Laboratory #14b confirmed the incorrect 186 keV counting-data handling in the questionnaire, and the other three did not respond to the question. In PG, apart from the highly overestimated results from laboratories #29 and #34, the other five remarkable deviations from the assigned value correspond to an underestimation of ^226^Ra activity concentration by laboratories #5, #9, #14a, #14b and #23. From these, laboratory #14b reported a void space above the sample material in the measurement container, and laboratory #9 assessed ^226^Ra through its progenies after only 7 days upon sealing of the container. Thus, the negative bias in their results indicates lack of secular equilibrium establishment between ^226^Ra and its progenies within the volume of the aliquot. Most laboratories correctly estimated the measurand values for both samples, but the performance scores worsened significantly when the precision of the results was considered. This attests to the general finding (§3.1) that laboratories had greater difficulty assessing the uncertainty of the measurand values rather than estimating these values with sufficient trueness. Positive biases were less common than negative biases, which may stem from lack of secular equilibrium establishment in the ^226^Ra subseries, as most laboratories determined ^226^Ra through its progenies. It is also noted that alpha spectrometry and liquid scintillation results were equivalent but not compatible to the gamma spectrometry results, with acceptable trueness but unacceptable precision due to uncertainty underestimation.

The reported activity concentration for ^214^Pb ranged from 59 to 598 Bq/kg for the PO and from 19 to 550 Bq/kg for PG. Bi-214 activity concentration was within a similar range for both materials (39 to 598 Bq/kg for the PO, and 13 to 672 Bq/kg in the PG). Most of the unacceptable results had negative bias, which may stem from the absence of equilibrium in the ^226^Ra subseries. This was evident in the results of participants #14a and #14b for PG, and #14a and #29 for PO that received action signals according to the respective z´, ζ and precision scores. Similarly to other NOR, laboratories’ performance dropped when the precision of the results was considered in addition to the other performance metrics.

The reported ^210^Pb activity concentration ranged from 126 to 670 Bq/kg for PO and from 179 to 768 Bq/kg for PG. Alpha spectrometry results (determination through ^210^Po assuming equilibrium) were equivalent to those of gamma spectrometry. Trueness of the results was generally acceptable, despite the objective complexity in the determination of this radionuclide due to the low energy of emitted photons and the relatively strong self-absorption phenomenon in NORM. Laboratory performance worsened significantly when the reported uncertainty was considered for the overall evaluation, as 13 out of 21 laboratories either received a warning signal for precision or had unacceptable precision scores for the PO, and 10 out of 21 for PG.

#### Members of the Thorium-232 decay series

**Fig. 8:**
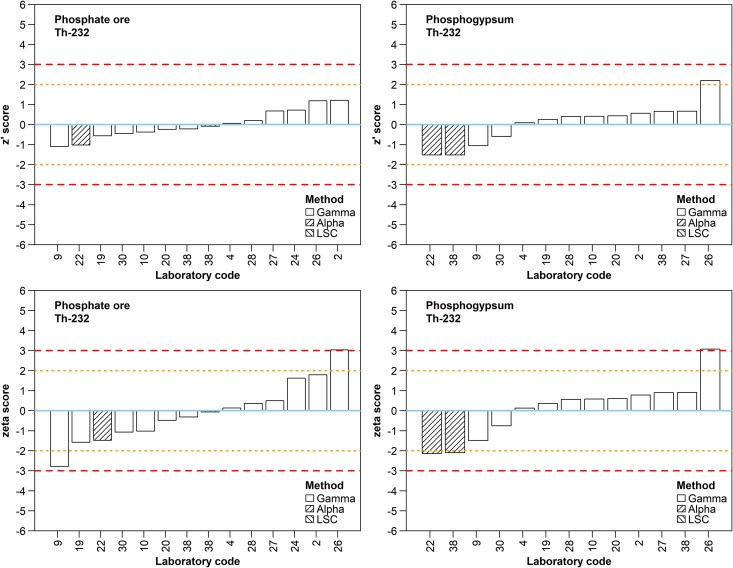
Z´ and ζ scores for ^232^Th by participant for the PO and PG samples. Refer to the caption of Fig. 4 for a description of the line patterns used in z´ and ζ score plots.

Apart from ^232^Th (Fig. 8), the z´ and ζ score plots for the members of ^232^Th decay series are presented as supplementary material (Fig. S9 to S13). The reported ^232^Th activity concentrations ranged from 36 to 52 Bq/kg for PO, and from 6 to 53 Bq/kg for PG. The range of the activity concentrations reported for the remaining members of ^232^Th decay series in PO was similar to that of ^232^Th (25 to 63 Bq/kg, overall, excluding ^208^Tl). The respective reported activity concentrations in PG ranged from 5 to 272 Bq/kg. The variability of the results and their biases from the assigned values were systematically higher for the PG sample, where disequilibrium within the series due to the PG production procedures was empirically expected and, eventually, seems to exist with high probability (see §2.4 and 3.1).

Overall, the performance of the participants on the determination of ^232^Th series’ members was assessed for 76 results reported for each material. The activity concentrations in PO were estimated with sufficient trueness, as only five results were unacceptable and another five received a warning signal. For PG, the most biased results were those of ^208^Tl and ^212^Pb. About 12 results were unacceptable in terms of trueness, five received a warning signal and the remaining were acceptable. From the above it can be concluded that the radiological characterization of PG was more difficult. The poorer performance could stem from incorrect handling of the equilibria between the subseries, the relatively high moisture content of PG, instability of the moisture content during the ICE, inconsistent methods for moisture determination among the participants, or inadequate consideration of these factors for the computation of the radionuclides’ activity concentrations on a dry mass basis, all of which causing a higher variability of the results. Of the 152 reported activity concentrations for radionuclides in the ^232^Th decay series, ~67 (44%) were deemed imprecise as they received “N” and “W” ratings for precision. Therefore, the previous issue regarding the correct estimation of the combined uncertainties occurs for members of the ^232^Th series too.

#### Activity concentration index I

The assigned value index *I* (*I_PT_*), its uncertainty *u*(*I_PT_*) and *σ_PT_* for PG were calculated with equation (1) using the assigned values for ^40^K, ^228^Ac, and ^226^Ra, as well as their uncertainties and the corresponding *σ_PT_* in quadrature. Note that there was no instruction for the participants to report *I*. Hence, the organizer calculated the potential results of index *I_i_* for PG, for each laboratory. In the absence of a reported result for ^232^Th, ^228^Ra activity concentration was considered for computing *I_i_*, followed by ^228^Ac and then ^228^Th. In the absence of ^226^Ra, ^214^Pb, or ^214^Bi were considered. A null value of ^40^K was adopted when its activity was not reported. Since PG is used as a superficial building material, it can be exempted from all restrictions concerning its radioactivity if *I* ≤ 2, therefore *E* ≤ 0.3 mSv·y^−1^, while for *I* > 6 the annual *E* would be higher than 1 mSv [20].

The overall pattern of *I_i_* is similar to that of ^226^Ra activity concentration, due to ^226^Ra having the highest activity concentration among the three radionuclides considered in the calculation, and thus having the highest contribution to *I*. All laboratories except two (#29 and #34) would be in the position to characterize PG as exempted with a confidence level >97%, since the upper limit of the 95% confidence interval of their result for *I* was below 2. Laboratory #29 could conclude that the material is not exempted but the potential annual effective dose (*E*) is within the range 0.3–1 mSv·y^−1^ with a confidence level > 97%, as the lower limit of the 95% confidence interval was higher than 2 but the upper limit was clearly below 6. Finaly, laboratory #34 could rate the radiological conformity of the PG as not acceptable at a confidence level > 97% since the lower limit of the 95% confidence interval of their result for *I* was significantly >6.

**Fig. 9:**
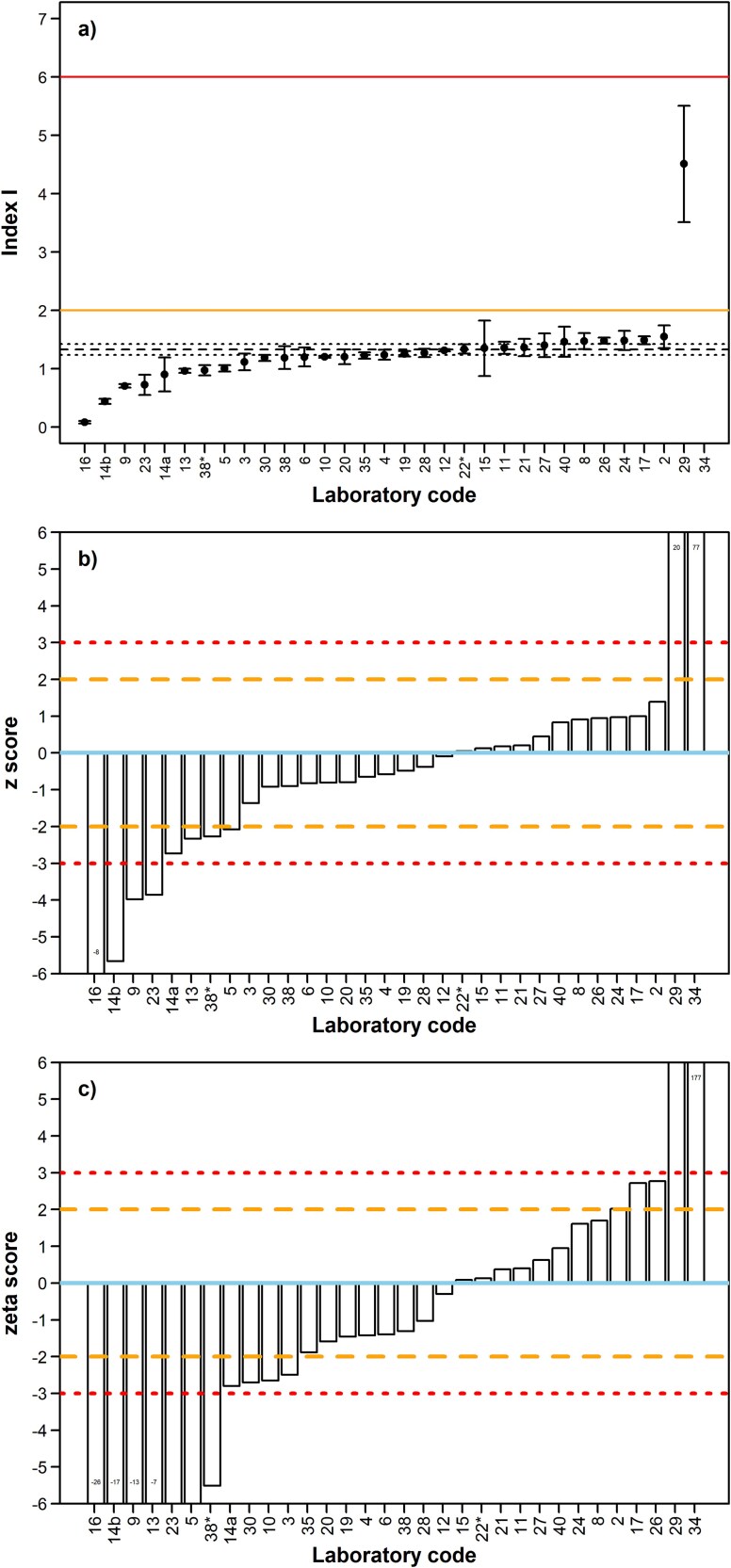
(**a**) Results of the index *I* for PG by participating laboratory and corresponding uncertainty for an expansion factor k = 2. The solid line at an Index I of 6 shows the limit that could result in a potential effective dose of more than 1 mSv·y^−1^ whereas the solid line at an Index I of 2 shows the exemption level (below which E ≤ 0.3 mSv/y). The black dashed and dotted lines correspond to the assigned value for index *I* (1.331) and its uncertainty (0.094) for k = 2. (**b**) Results for z scores. Refer to the caption of Fig. 4 for a description of the line patterns used in z´ and ζ score plots. Laboratories #16, #29 and #34 obtained an absolute z score >6, which has been intentionally excluded from complete visual representation to avoid distorting the scale. (**c**) Results for ζ scores. Refer to the caption of Fig. 4 for a description of the line patterns used in the plot. Laboratories #5, #9, #13, #14b, #16, #23, #29, and #34 obtained an absolute ζ score >6, which has been intentionally excluded from complete visual representation to avoid distorting the scale. An asterisk after the laboratory code indicates ^232^Th activity concentration was estimated with radiochemical methods involving alpha spectrometry.

The underlying reason for the variability of Index *I* with a clear predominance for a negative bias is connected to the underestimation of ^226^Ra activity concentration by most participants, likely due to lack of equilibrium between ^226^Ra and its progenies within the aliquot. Index *I* results higher than 1.644 and lower than 1.017, accounting for 32% of the participants, were considered unacceptable or given warning signals according to z scores (Fig. 9b). Performance according to ζ scores was worse, as ~52% of the laboratories were given warning or action signals (Fig. 9c).

## Discussion

Measurements were performed by the laboratories using their routine methodology and procedures as instructed by the organizer; newly introduced or even short-term tested/applied procedures were out of ICE scope and objectives. Consequently, there was considerable diversity in e.g. sample preparation, measurement procedures, data processing, etc., which provides valuable insights into the current laboratory practice in the region. A summary of these practices is attached as Supplementary material (Table S3).

###  

#### Moisture determination

Moisture determination was typically carried by desiccation of subsamples with varying masses (ranging from 2 to 100 g), at different temperature intervals (ranging from 40°C to 105°C), over designated durations (ranging from 4 hours to 2 days) or unspecified periods (e.g. a few hours, overnight, or until a constant mass was achieved). The wide range of moisture values reported for PG may be justified by the large variety of these practices, since the dehydration of PG depends strongly on both temperature and time (e.g. [22]).

#### Sample preparation for gamma spectrometry

Certain laboratories filled the gamma-spectrometry containers directly with the provided samples (#2, #5, #8, #9, #11, #12, and #19), while others implemented different procedures, including drying, milling, or sieving the samples. Many laboratories recognized the importance of taking actions to achieve the best possible accuracy and compatibility of the results, such as filling the measurement containers in a way to prevent a void air space above the sample matrix where radon progenies can accumulate [23]. About half of the laboratories explicitly indicated filling the containers to maximum capacity or without leaving, by other means, any air pocket above the aliquot (#3, #4, #6, #9, #10, #11, #13, #15, #17, #21, #22, #24, #28, #30, and #40). For example, participant #24 covered the aliquots’ material surface with a polymer-coated aluminum foil and filled a sufficiently thick portion of the void volume with an epoxy resin highly impermeable to radon. On the other hand, laboratories #2, #12, #14b, #16, #27, #29, #35, and #38 specified a partial container filling approach.

The geometry, volume and composition of the containers were varied, with certain laboratories employing Marinelli beakers, while others opted for cylindrical and planar jars (Table S3), featuring materials such as metal, polypropylene, acrylic, polyvinyl chloride and polyethylene. Although most container materials are suitable for gamma spectrometry, attention to radon diffusion through certain polymers is necessary to achieve the best possible accuracy of ^214^Pb, ^214^Bi, and ^226^Ra results as the radon diffusion coefficients may differ by several orders of magnitude across different polymers (e.g. [24, 25]). Bonczyk & Samolej [26] have also suggested that cylindrical containers are less prone to radon leakage compared to Marinelli beakers. While the container material and shape can significantly affect radon permeability, poor sealing of the container lid is often the main cause of radon leakage. The questionnaires showed that homogenization of the sample materials and sealing of the containers were routine procedures among participants. Sealing, which is necessary for ^226^Ra subseries’ equilibrium establishment within the sample container, included the use of tape, aluminum foil, polymer-coated aluminum sheet in combination with epoxy resin, silicone, glue, bee wax, vacuum grease, parafilm, epoxy resin and a combination of epoxy resin and varnish. However, it was not clear if all these sealants and sealing procedures fulfilled their objective and to what extent, since only five laboratories (#3, #17, #24, #27, and #40) confirmed the radon-tightness of their measurement containers through testing. The positive biases of ^214^Pb and ^214^Bi or ^226^Ra results reported by these five participants, indicate that epoxy and silicone-based resin sealants were less permeable to radon. In fact, this has been previously demonstrated (e.g. [27, 28]). Unfortunately, most procedures for testing the radon tightness are somewhat tedious and time consuming, and therefore often ignored. It appears that this was also the case in present ICE. From the participants who did not test for radon leakage, the highest negative biases for both samples belong to the two laboratories that used glue and vacuum grease (#9 and #13), possibly due to the nature of these sealing materials. This speculation is supported by the fact that radon is highly soluble in many organic compounds including greases [29, 30]. Hence, radon could be adsorbed onto the sealant outside the sample volume and potentially escape from the container through it.

The majority of the participants stored the sealed samples for extended time (ranging from 7 days to 3 months), to attain radioactive equilibrium within ^226^Ra and ^228^Th subseries prior to measurement. One laboratory (#9) determined the activity of ^226^Ra through its progenies as many participants did, however, the period between sealing the container and measurement lasted only 7 days, fact which could be one of the possible reasons of underestimation of radium activity concentration in both materials, leading to an unacceptable result for PG in terms of trueness. Most of the unacceptable results for ^214^Pb and ^214^Bi were also found to have negative biases, which might be related to the absence of secular equilibrium establishment, caused by counting the samples before the required time for parent-daughters activity equalization, lack of radon-tightness, a void space above the material aliquot in the measurement container or a combination of the previous situations.

#### Sample preparation for alpha spectrometry

Three laboratories reported results determined by alpha spectrometry (#9, #22 and #38). One of them spiked their samples with ^232^U and ^229^Th tracers and performed extraction chromatography (#22), whereas a second one declared the extraction of specific radionuclides by radiochemical isolation without applying any kind of chromatography (#9). The chemical recovery was >72% in all cases and counting time ranged from 2000 s to 25 000 s. Their results were generally equivalent to the respective ones from gamma spectrometry except for ^232^Th in the PG sample. In this case, the activity concentration results from alpha spectrometry were significantly lower than those estimated through ^232^Th progenies using gamma spectrometry. Given the higher specificity of alpha spectrometry for ^232^Th determination, the above provides compelling evidence that secular equilibrium within the ^232^Th decay series was not established in the PG sample. In cases where equilibrium status evaluation is difficult and the date of last modification of a material is unknown or cannot be determined through Bateman equation [5, 6], laboratories should not report results for radionuclides such as ^232^Th, ^238^U or ^210^Po, unless they use alpha spectrometry-based methods. On these grounds, laboratory #24 did not report ^232^Th activity concentration for the PG sample, since they did not perform α-spectrometry but they clearly detected disequilibrium between ^228^Ra and ^228^Th -i.e. within the ^232^Th series—through HRGS. On the other hand, the laboratories that used ^232^Th progenies to assess its activity concentration, significantly overestimated ^232^Th activity concentration in PG (#2, #4, #9, #10, #19, #20, #26, #27, #28, and #30). However, unacceptable results (N) for ^232^Th were absent, a fact that is related to the common bias of the ^232^Th assigned value for PG, since it is likely that the only accurate results for ^232^Th were the ones provided by laboratories using alpha spectrometry (#22 and #38).

#### Sample preparation for liquid scintillation analysis

One laboratory (#15) performed two-phase LSC to determine ^226^Ra in PO. To this purpose, an aliquot of the sample was digested and dissolved in HNO_3_ and water to form an aqueous solution to which a water-immiscible organic scintillator was added in a 20 ml polypropylene LSC vial (1:1 sample to cocktail ratio). Ra-226 activity concentration was estimated through members of the ^222^Rn subseries partitioned in the organic phase (Cantaloub, 1997). The result was acceptable in terms of trueness, but the uncertainty was severely underestimated and must be revised.

#### Gamma spectrometry measurement

All the participants performed HRGS using high purity germanium detectors (HPGe) with variable counting durations, averaging 169 000 s. Most of the results obtained with “large planar” detectors were of acceptable accuracy (87% for PO and 82% for PG). The percentage of acceptable results in terms of accuracy when either “reverse electrode coaxial” or “thin entrance electrode coaxial” detectors were used was lower (78% for PO and 75% for PG) and dropped further when “standard electrode coaxial” detectors were employed (to 74% and 69%, respectively). The higher acceptance rates in terms of accuracy when “large planar” detectors were used, could be attributed to the combination of the facts that the majority of the participants packed the aliquots of the two ICE items in cylindrical/planar sample geometry, while these detectors exhibit optimum spectral characteristics -including their enhanced energy response—in the energy range that is most important for the determination of all the γ-emitting NOR in NORM, when samples of such geometry are counted close to the detector endcap [31, 32].

#### Calibration

The responses to the questionnaire highlighted a variety of calibration approaches, involving the use of materials with known activity concentrations, efficiency transfer, Monte Carlo simulation, specialized software, and combinations of the previous methods, equally split. According to ISO 20042 generic test method for HRGS [10], all these methods are metrologically sound, and therefore acceptable since they can provide equivalent results traceable to the SI. Nevertheless, attention must be paid to the computational procedures, which have become increasingly popular but require considerable expertise.

#### Data processing and assumptions

Most participants (at least 22 out of 31) made assumptions on the equilibrium status of the (sub)series for the estimation of the activity concentration of parent radionuclides such as ^238^U, ^226^Ra, ^232^Th, ^228^Ra, and ^228^Th in the test items. As discussed earlier, for equilibrium to become established within some subseries (e.g. ^226^Ra and ^228^Th), appropriate sample preparation, a well-sealed container and sufficient waiting time are required.

For one of the ICE items or both of them, a few laboratories also referred to the assumptions and methodology behind the estimation of ^226^Ra and/or ^235^U through the 186 keV γ-peak, needed due to overlapping of ^226^Ra and ^235^U, when the activity concentration of one of these radionuclides was not determined directly by more specific measurements (e.g. through α-spectrometry). In this case, the signal due to the activity of one of the two radionuclides is calculated indirectly and subtracted from the 186 keV area; then, the remaining net area is used for the determination of the activity of the second radionuclide: For the determination of ^226^Ra, ^235^U can be calculated exclusively either (i) through the world average ^238^U/^235^U activities’ ratio and the measured ^234^Th activity concentration, assuming equilibrium between ^238^U and ^234^Th; or (ii) through the activities of ^235^U-daughters, assuming equilibrium within ^235^U series in the aliquot; or even (iii) through the weighted mean activities resulting from both (i) and (ii). Similarly, for the determination of ^235^U, the signal in 186 keV peak due to ^226^Ra activity can be calculated exclusively through the activities of its progenies ^214^Pb/^214^Bi, after equilibrium establishment for ^226^Ra subseries within the sealed aliquot. Most laboratories determined ^226^Ra activity concentration by its progenies only. A few laboratories determined ^226^Ra exclusively by its 186 keV line (#22, #29 and #38), whereas others considered both ^226^Ra progenies and the 186 keV line (#9, #16, #24 and #40). From the latter laboratories, one (#16) did not apply any corrective action to resolve the interference. Most of the participants who determined ^235^U through the 186 keV peak also accounted for the overlap with ^226^Ra. Only two laboratories calculated ^235^U exclusively through the measured ^238^U/^234^Th activity concentration and the world average ^238^U/^235^U ratio (#13 and #38).

Finally, more than 10 participants stated secular equilibrium assumptions pertaining to decay subchains other than ^226^Ra and its short-lived daughters, such as between ^238^U and ^234^Th, ^232^Th and ^228^Ra/^228^Ac or ^228^Ra and all its progenies. About 14 laboratories reported results for ^238^U. Thirteen of them determined ^238^U assuming secular equilibrium with its gamma-emitting progenies ^234^Th and ^234m^Pa, using the 63 keV and (in a few cases) 1001 keV lines, respectively. One laboratory (#24) calculated ^238^U as the weighed mean of the activities which were measured individually by the ^234^Th gamma peaks at 63, 93 (doublet) and 113 keV (after correction for ^227^Th interference). This participant intentionally avoided to use also the activity derived by the 1001 keV peak of ^234m^Pa due to the high variability found in literature and the still possible inaccuracy of this line’s intensity values as currently provided by well-known nuclear data sources [33–35].

In this ICE, ^232^Th activity concentration was mainly estimated from its gamma-emitting progenies, considering the activity concentration of ^228^Ra, ^228^Ac, both ^228^Ra and ^228^Th, ^228^Ac and ^212^Pb and other combinations including ^208^Tl. It is also worth noting that most laboratories which reported ^228^Ra determined its activity concentration through ^228^Ac, given secular equilibrium between ^228^Ra and ^228^Ac is established within two days after any chemical disturbance. Th-228 was determined either by direct measurement of ^228^Th using α-spectrometry or through different combinations of its progenies (^212^Pb, ^212^Bi, ^208^Tl, and ^224^Ra).

Th-232 determination using gamma-spectrometry is one of the most debated issues in NORM characterization. This nuclide is one of the three parameters considered in the widely used activity concentration index *I* for the radiological control of building materials (see eq. 1). However, as a practically pure α-emitting radionuclide, ^232^Th cannot be measured directly by gamma-spectrometry, but by more specific radioanalytical techniques, such as alpha spectrometry-based methods, which are not widely available or cost effective for regular radiological characterization of building materials. Hence, ^232^Th concentration is usually estimated using the findings from the short-lived gamma-emitting progenies of ^228^Ra and ^228^Th. When ^228^Ra and ^228^Th are in secular equilibrium, a standard convention is to calculate the activity concentration of ^232^Th as the weighted mean of the fully supported ^228^Ra/^228^Ac and ^228^Th/^212^Pb concentrations. However, when the material has undergone industrial or chemical processing, the ^232^Th decay series might be permanently out of secular equilibrium [36] and the above convention is no longer applicable. When a reliable determination of ^232^Th is impossible, its activity concentration in the index *I* formula can be replaced by the activity concentration of ^228^Ra or ^228^Ac [37]. Ra-228 progenies are the major contributors to the external radiation exposures from ^232^Th decay series contained in building materials, therefore the above replacement is justified from a radiation protection point of view and considered legally acceptable. Alternatively, if there is disequilibrium, the activity concentration index *I* can be calculated using the highest of the ^228^Th and ^228^Ra concentrations [38]. The use of ^232^Th over ^228^Ra is justified if its concentration is higher, since ^228^Ra activity concentration is expected to increase until it equalizes the activity of its parent nuclide upon secular equilibrium establishment. Nevertheless, guidance on this matter is welcomed, to ensure consistency in the assessment of dose from these types of materials.

#### Quality management system

Nineteen laboratories are accredited for gamma-spectrometry measurements (Table S3), mostly under ISO 17025. Two laboratories do not have accreditation but are authorized/recognized by their country’s regulatory bodies to perform characterization of NORM. Of the unaccredited laboratories, five have implemented a QMS, four do not have one in place and three did not provide any information on QMS or accreditation status. Of the participants who reported alpha spectrometry results, two are accredited and implementing a QMS.

About 29% of the participants had acceptable accuracy for all the results they reported (namely, laboratories #2, #4, #8, #20, #21, #24, #28, #35, and #40). Of these, three laboratories (33%) are accredited, another three implement a QMS and the remainder declared the absence of adherence to such a system. From an alternative point of view, only 16% of the accredited participants characterized both materials with sufficient accuracy (regarding all the radionuclides of interest they reported), while, in contrast, this happened for 50% of the unaccredited laboratories. This could be caused by a more relaxed behavior of accredited laboratories due to “proven” analytical quality or to a greater caution and focus or greater experience of non-accredited laboratories, thus obtaining better analytical results. However, the conducted questionnaire did not provide sufficient evidence to draw conclusions about these aspects. It is important to note that accreditation is not mandatory for radiological characterization of NORM in some of the countries whose laboratories participated in this ICE. In this context, some laboratories choose not to obtain accreditation, as it is an expensive process, which raises the per-sample analysis costs for end users and leads to fewer analyses, failing to adequately address society’s safety needs. The results obtained in this ICE show that the majority of the unaccredited laboratories have implemented rigorous quality assurance practices, which allowed them to obtain good results. Moreover, accreditation alone should not be considered as indisputable evidence of high analytical quality and reliability. Even though accreditation is acknowledged as playing an important role towards quality improvement, the performance of the radioanalytical laboratories in a series of recent ICEs or PTs, like the ones organized annually by highly recognized and metrologically reliable entities such as the IAEA, holds significant importance and value.

## Conclusions

The joint IAEA/EEAE intercomparison exercise involved the radioanalytical characterization of PO and PG samples by over thirty laboratories. All the laboratories applied (at least) high resolution gamma-ray spectrometry for the characterization of the samples, showing different degrees of success in meeting the target measurands’ values and assessing their uncertainties. It was observed that laboratories performed generally well, in spite of applying different practices regarding sample preparation, sealing techniques, measurement geometry and calibration procedures. Participants’ performance on the precision of the results was almost always worser than their performance on trueness and compatibility, highlighting the need for improvement on combined measurement uncertainty estimation.

The results highlighted differences in the activity concentrations of the radionuclides between the two sample types, with PO generally exhibiting higher concentrations compared to PG. The impact of technological processing on the state of radioactive equilibria between the members of each natural series in the materials was also clearly identifiable through the differences in the activity concentrations estimated by the participants. Disequilibria were evident in the PG ICE item, while in the unprocessed PO the equilibria were not disturbed, as was theoretically expected. However, not all participants in the ICE handled this subject in an appropriate manner, therefore the present study focused on these issues with added emphasis. Most laboratories estimated ^226^Ra and ^232^Th activity concentrations through the measurement of their short-lived progenies assuming secular equilibria in the respective decay series, however, only a few laboratories conducted tests to validate these assumptions. According to the above, it is clear that, if the assigned values cannot be provided by external metrologically advanced entities, their generation based on carefully selected rational results of a subgroup of participants applying well documented good laboratory practices, such as validation tests, is justified and should be always preferred over the use of the entire results’ population. Considering that the radionuclides directly measured are not always the ones used for hazard estimation, we argue that ICEs focused on NORM characterization based on a two-round exercise seem justified. Firstly, participating laboratories would be asked to provide results for radionuclides directly measured by applying their routine methodology and procedures and without additional information about the test sample. These results would be used for the evaluation of proficiency and accuracy of the participating laboratories. In a second phase, additional information about the test sample, such as its history, would be provided to the laboratories, which would be asked to interpret their initial results to get information about the long-lived radionuclides that are not directly measured or to correct their initial results based on the necessary assumptions.

Overall, the findings from this IC exercise provide valuable insights for enhancing laboratory practices and methodologies in the measurement of NORM, such as ensuring the homogeneity of the aliquot and that the equilibrium assumptions made in the determination of NORs are met (more details are discussed in the Supplementary material, section A2). These insights will ultimately contribute to improved quality assurance in radiation monitoring and dose assessment. Ensuring adherence to established standards is widely recognized as crucial for maintaining the accuracy and reliability of NORM analyses. Interestingly, this study showed that accreditation status does not necessarily correlate with analytical accuracy, as most of the unaccredited laboratories demonstrated equivalent or better performance compared to the accredited ones. Hence, the present study emphasizes that the performance of the radioanalytical laboratories in recent proficiency testing or analytical quality assessment schemes organized by globally recognized and metrologically reliable entities, such as the IAEA, serves as the basic proof of their current analytical quality and capabilities. This evidence should be legally recognized and utilized accordingly.

## Supplementary Material

Figure_S1_ncaf003

Figure_S2_ncaf003

Figure_S3_ncaf003

Figure_S4_U-238_ncaf003

Figure_S5_Th-234_ncaf003

Figure_S6_Pb-214_ncaf003

Figure_S7_Bi-214_ncaf003

Figure_S8_Pb-210_ncaf003

Figure_S9_Ra-228_ncaf003

Figure_S10_Ac-228_ncaf003

Figure_S11_Th-228_ncaf003

Figure_S12_Pb-212_ncaf003

Figure_S13_Tl-208_ncaf003

Supplementary_material_Appendix_1_Table_S1_ncaf003

Supplementary_material_Appendix_2_Table_S2_ncaf003

Supplementary_material_Appendix_3_Table_S3_ncaf003

Fig_ncaf003

## Data Availability

Data is provided as supplementary material.
