# Supplementary figures and images for: Results of the joint IAEA/EEAE Intercomparison exercise on radioanalytical characterization of NORM samples in the European region
Source: Radiat Prot Dosimetry. 2025 Feb 20;201(3):223–46. doi: 10.1093/rpd/ncaf003 (PMC11884514; doi:10.1093/rpd/ncaf003)

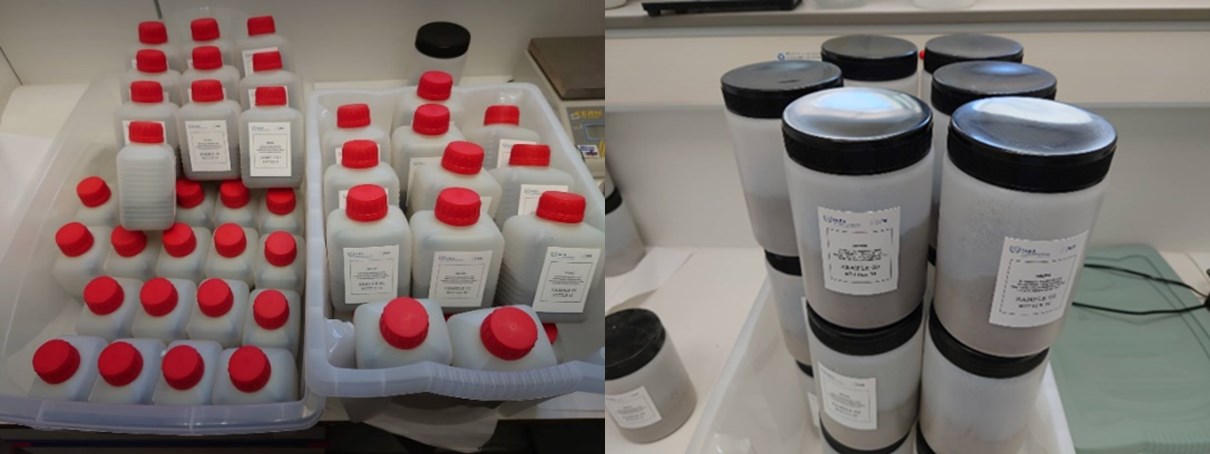

Supplement: Figure_S1_ncaf003 [file figure_s1_ncaf003.jpeg]

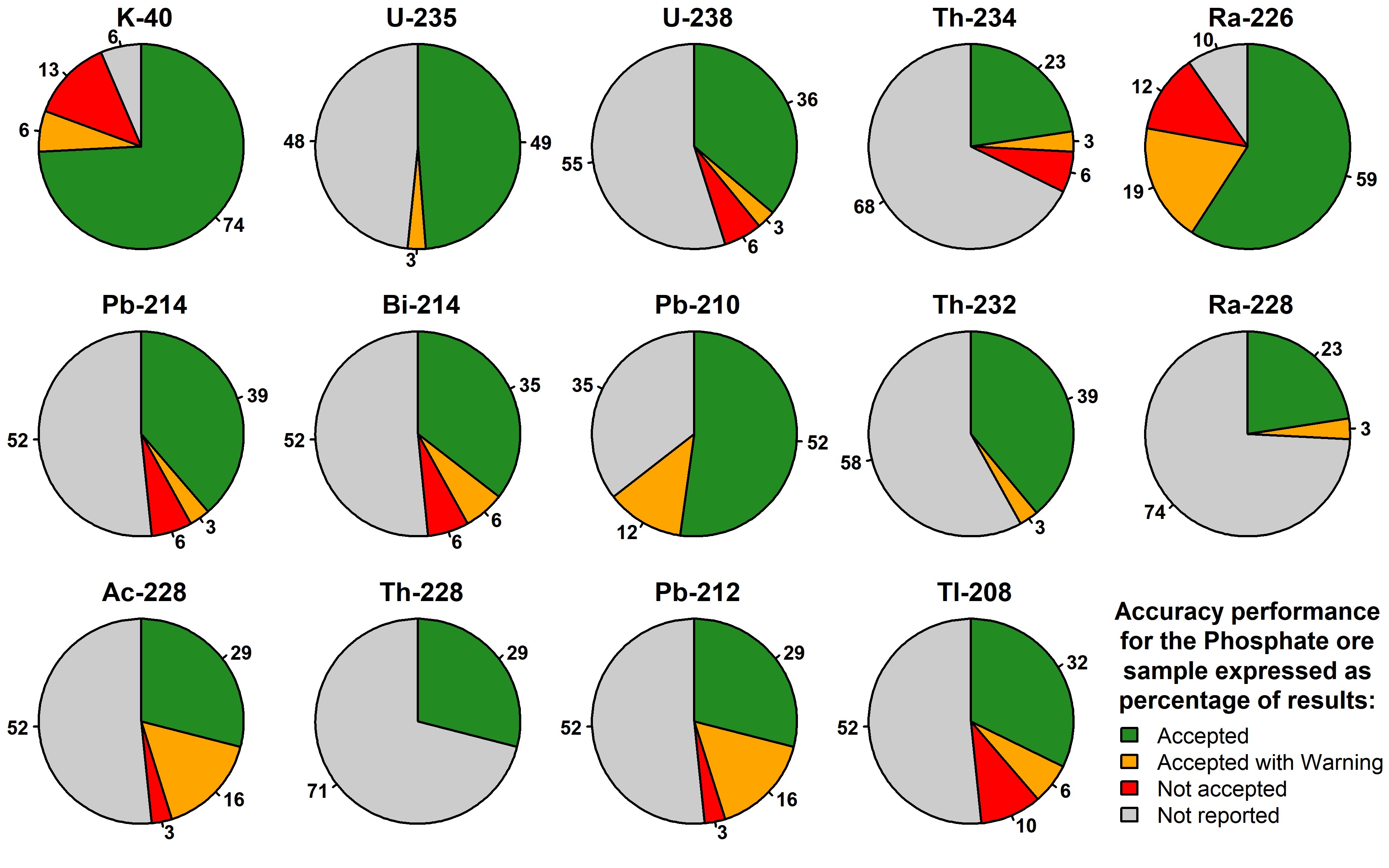

Supplement: Figure_S2_ncaf003 [file figure_s2_ncaf003.jpeg]

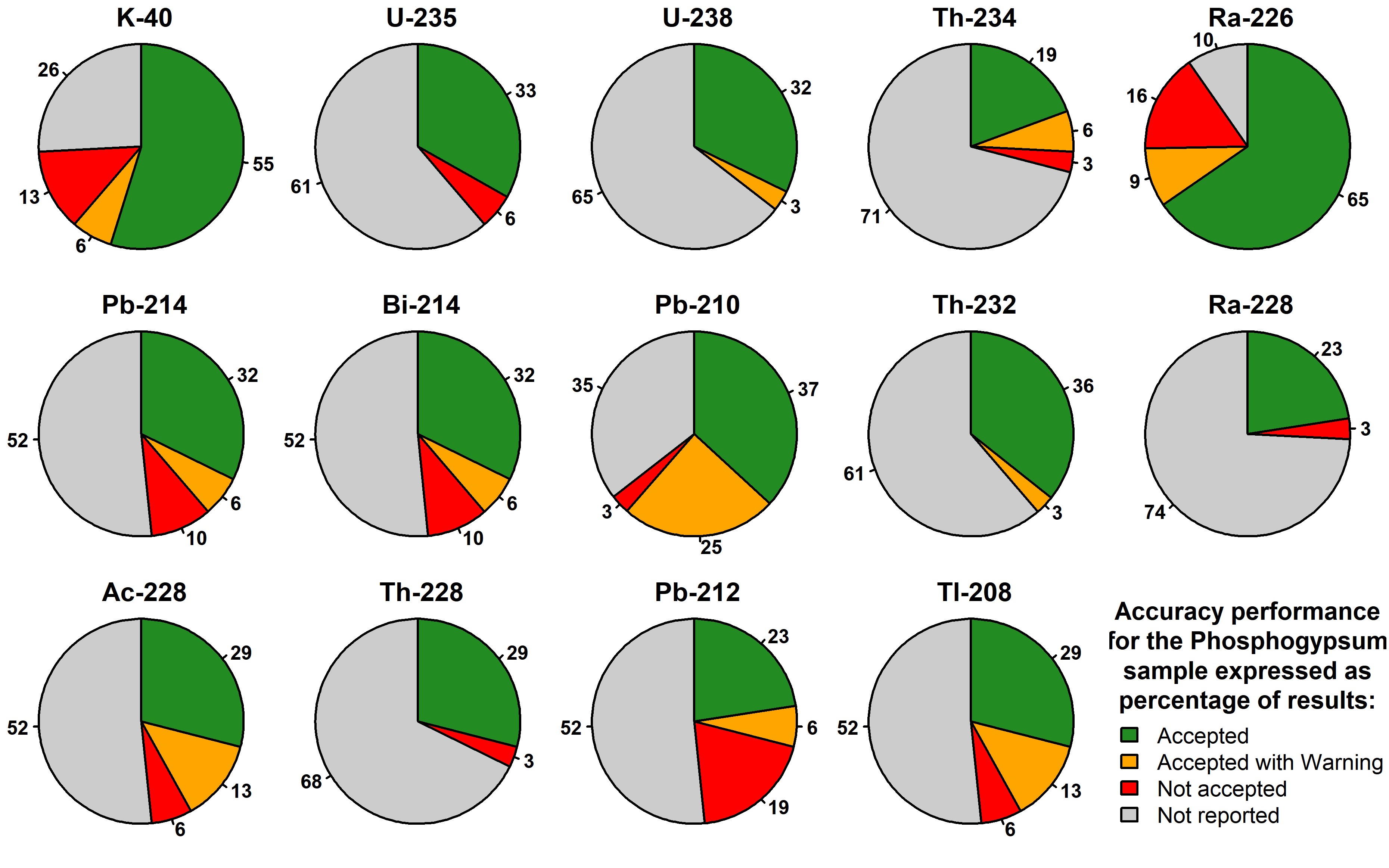

Supplement: Figure_S3_ncaf003 [file figure_s3_ncaf003.jpeg]

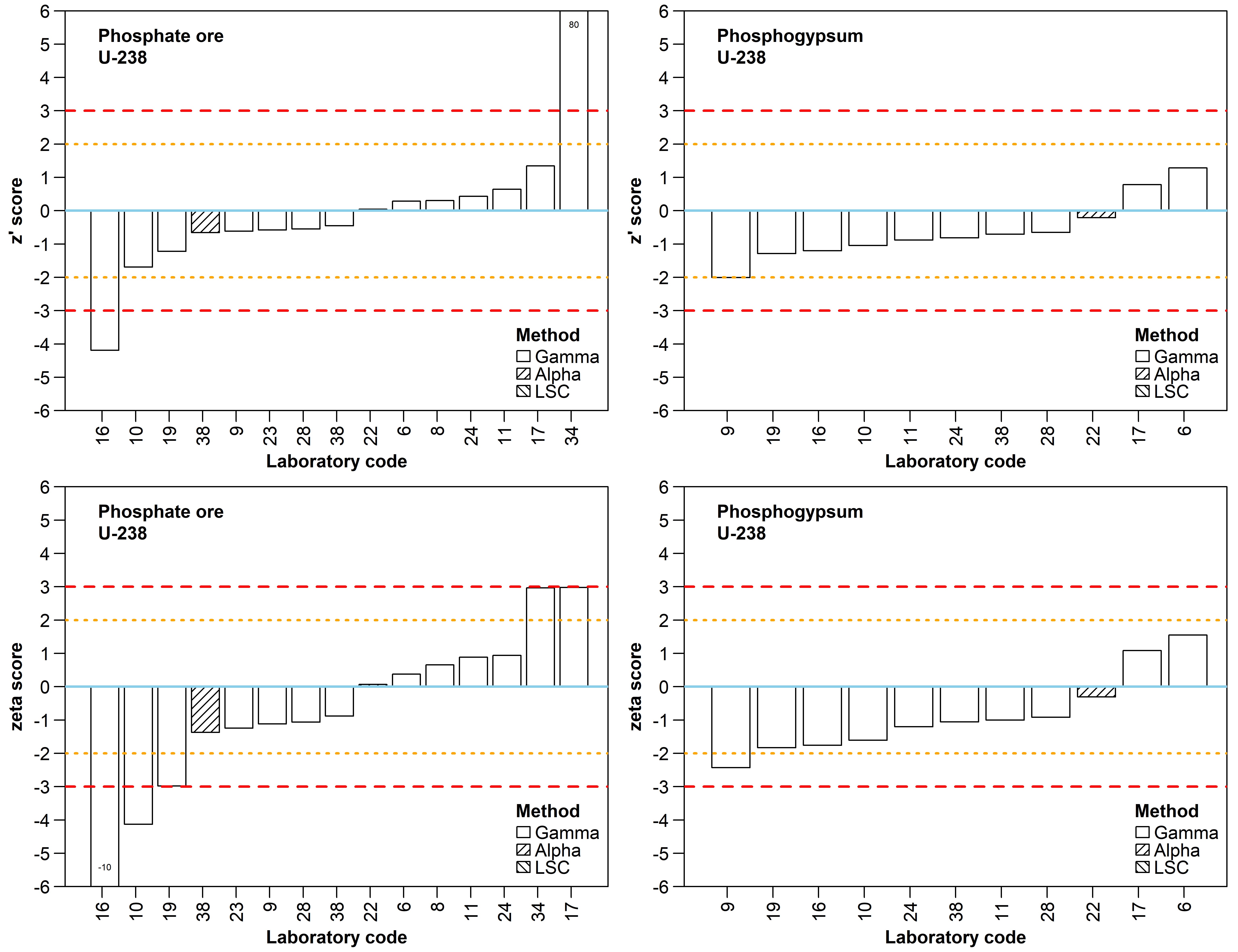

Supplement: Figure_S4_U-238_ncaf003 [file figure_s4_u-238_ncaf003.jpeg]

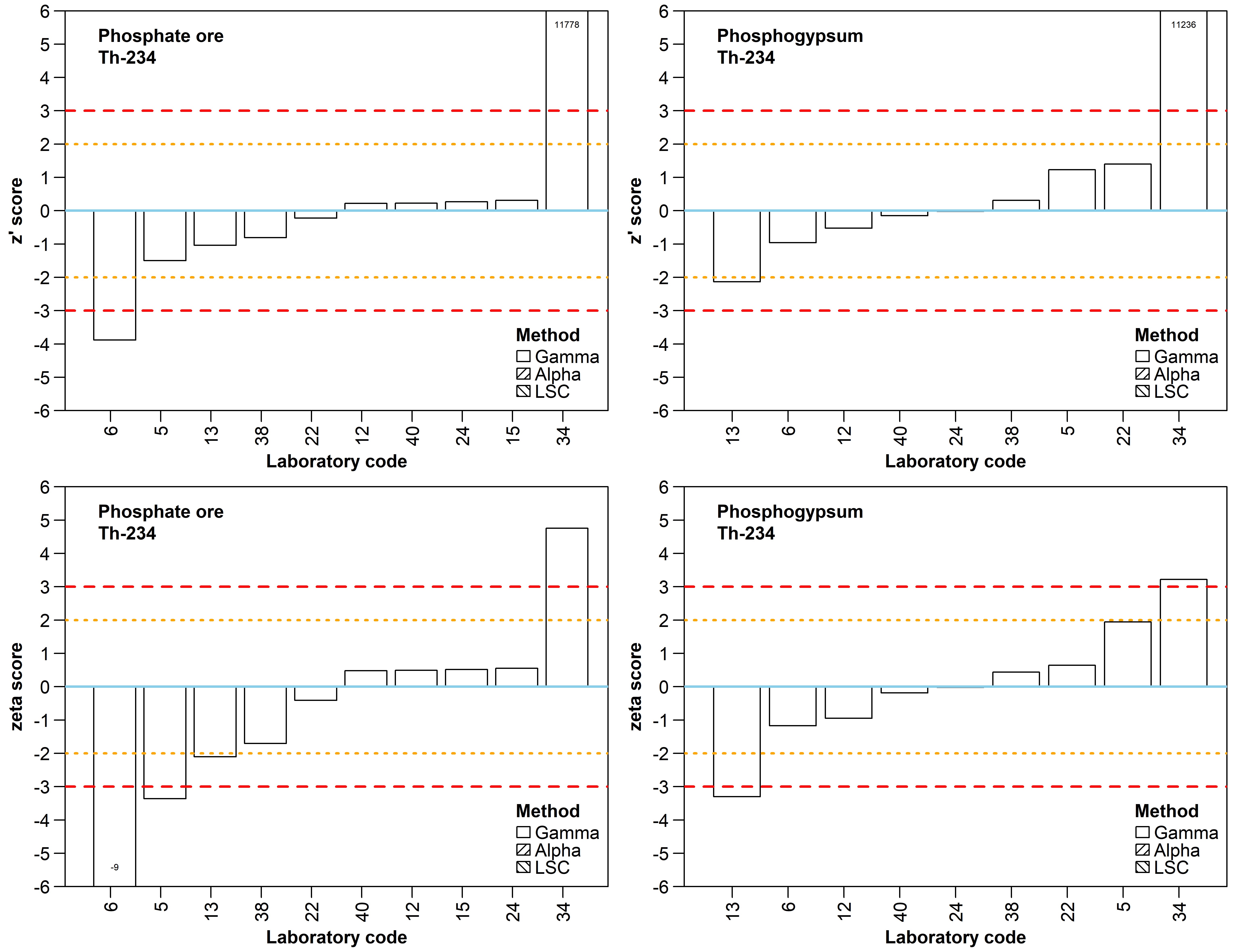

Supplement: Figure_S5_Th-234_ncaf003 [file figure_s5_th-234_ncaf003.jpeg]

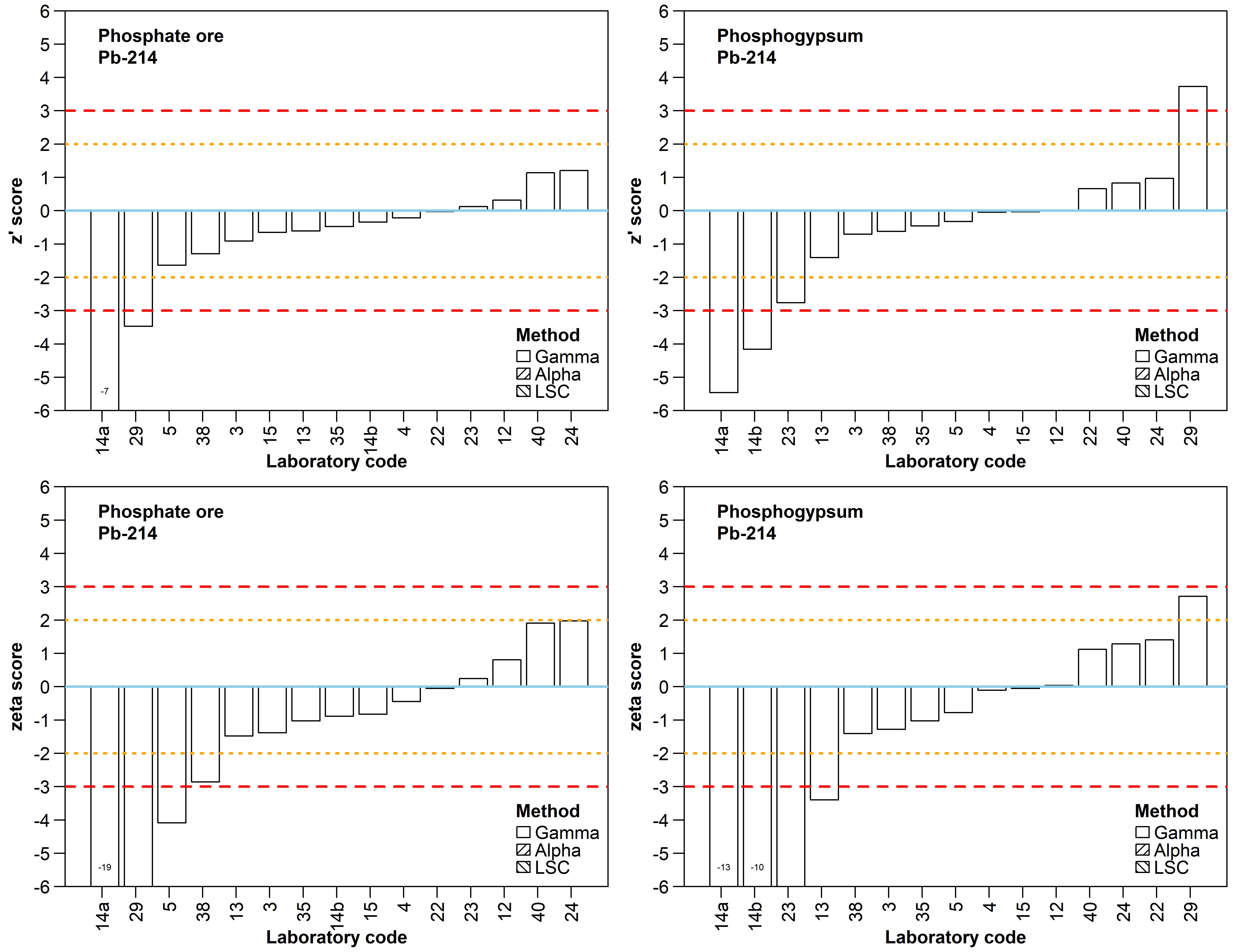

Supplement: Figure_S6_Pb-214_ncaf003 [file figure_s6_pb-214_ncaf003.jpeg]

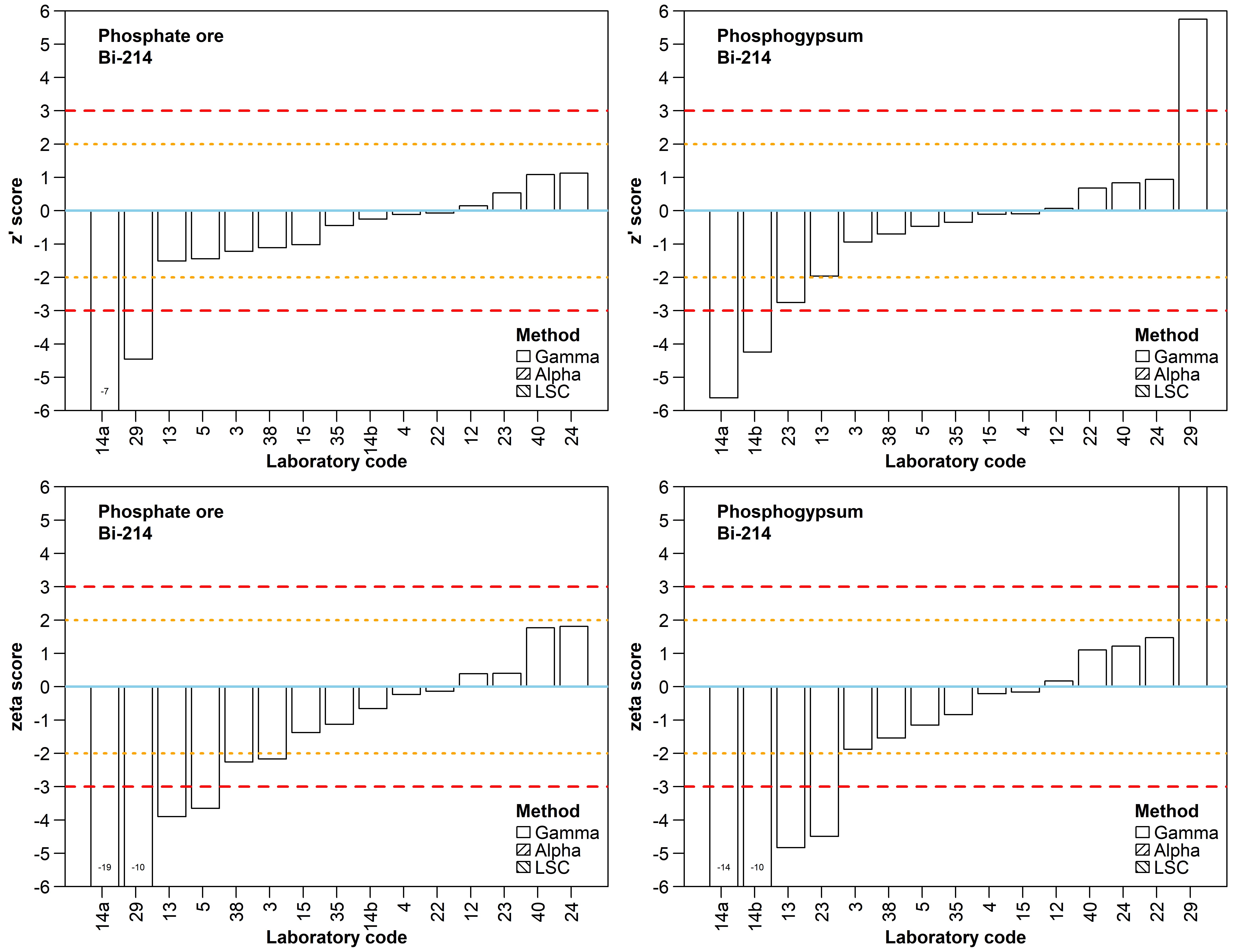

Supplement: Figure_S7_Bi-214_ncaf003 [file figure_s7_bi-214_ncaf003.jpeg]

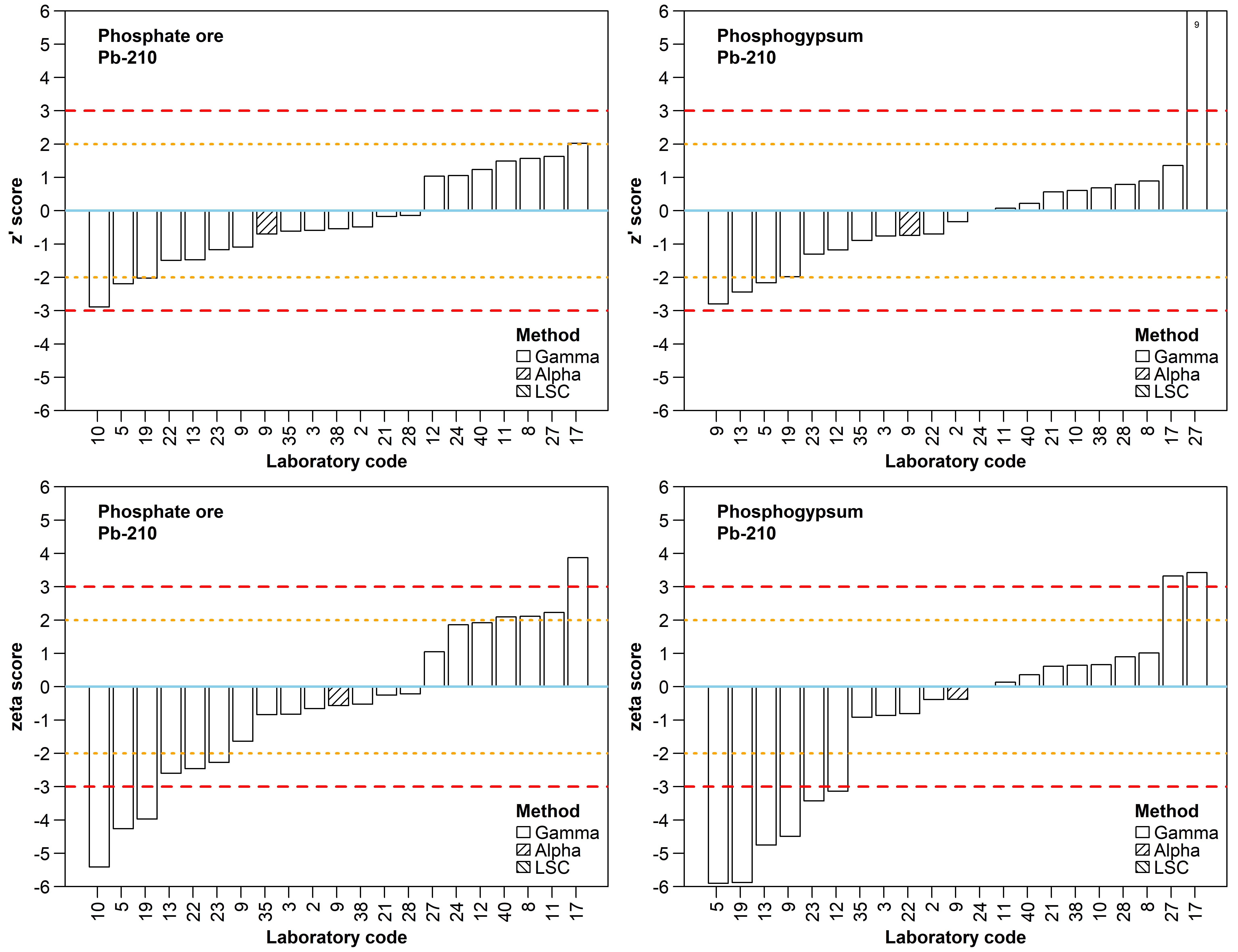

Supplement: Figure_S8_Pb-210_ncaf003 [file figure_s8_pb-210_ncaf003.jpeg]

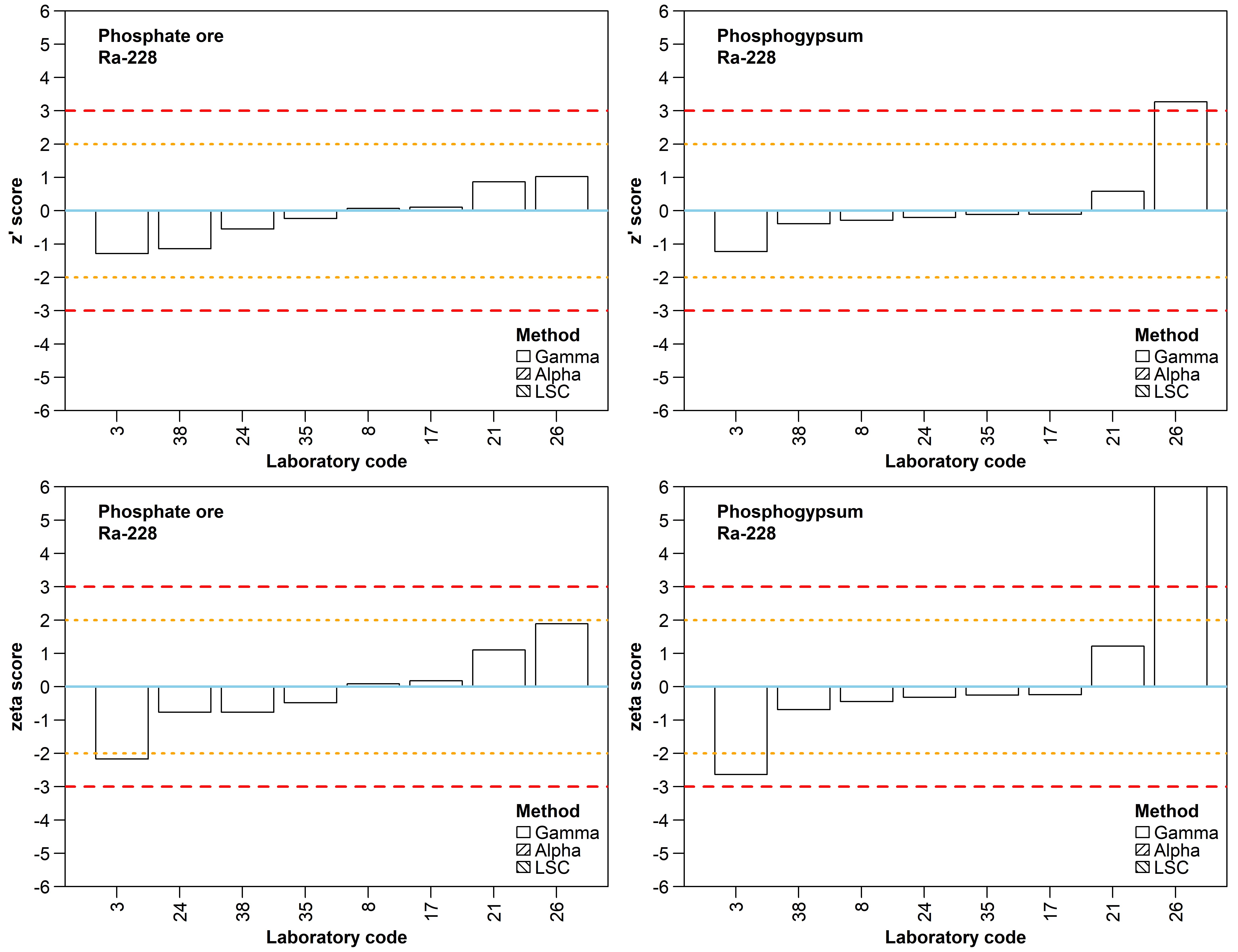

Supplement: Figure_S9_Ra-228_ncaf003 [file figure_s9_ra-228_ncaf003.jpeg]

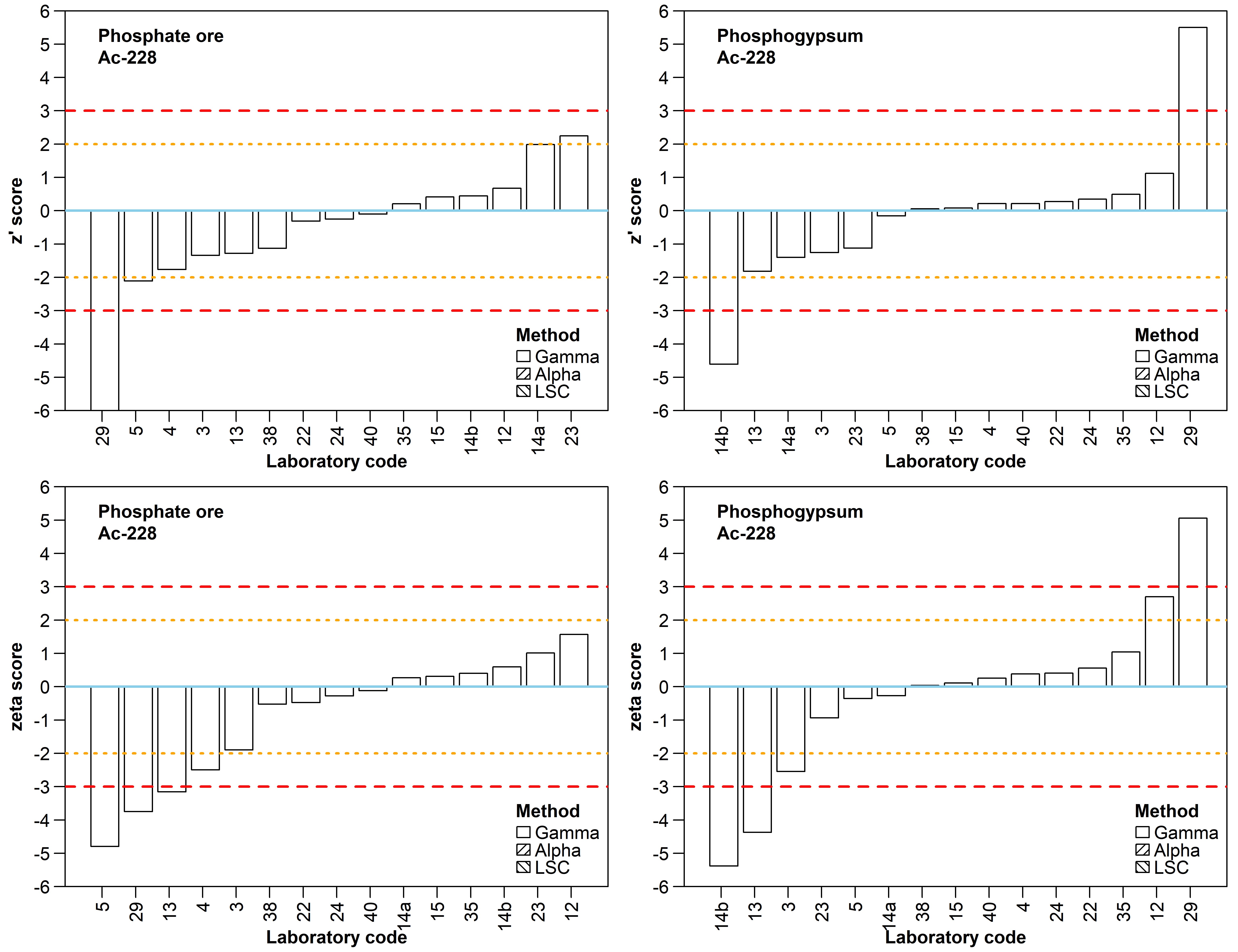

Supplement: Figure_S10_Ac-228_ncaf003 [file figure_s10_ac-228_ncaf003.jpeg]

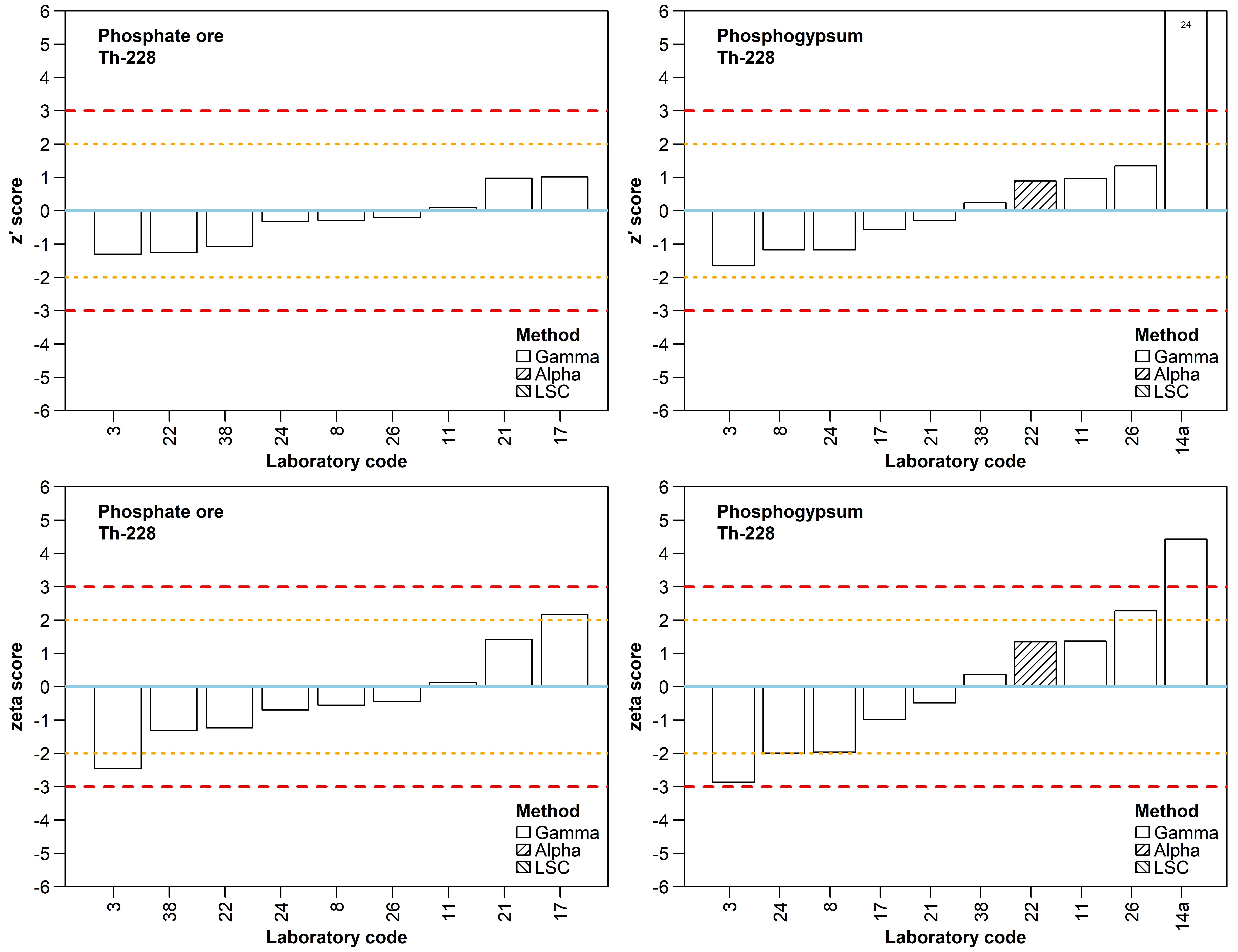

Supplement: Figure_S11_Th-228_ncaf003 [file figure_s11_th-228_ncaf003.jpeg]

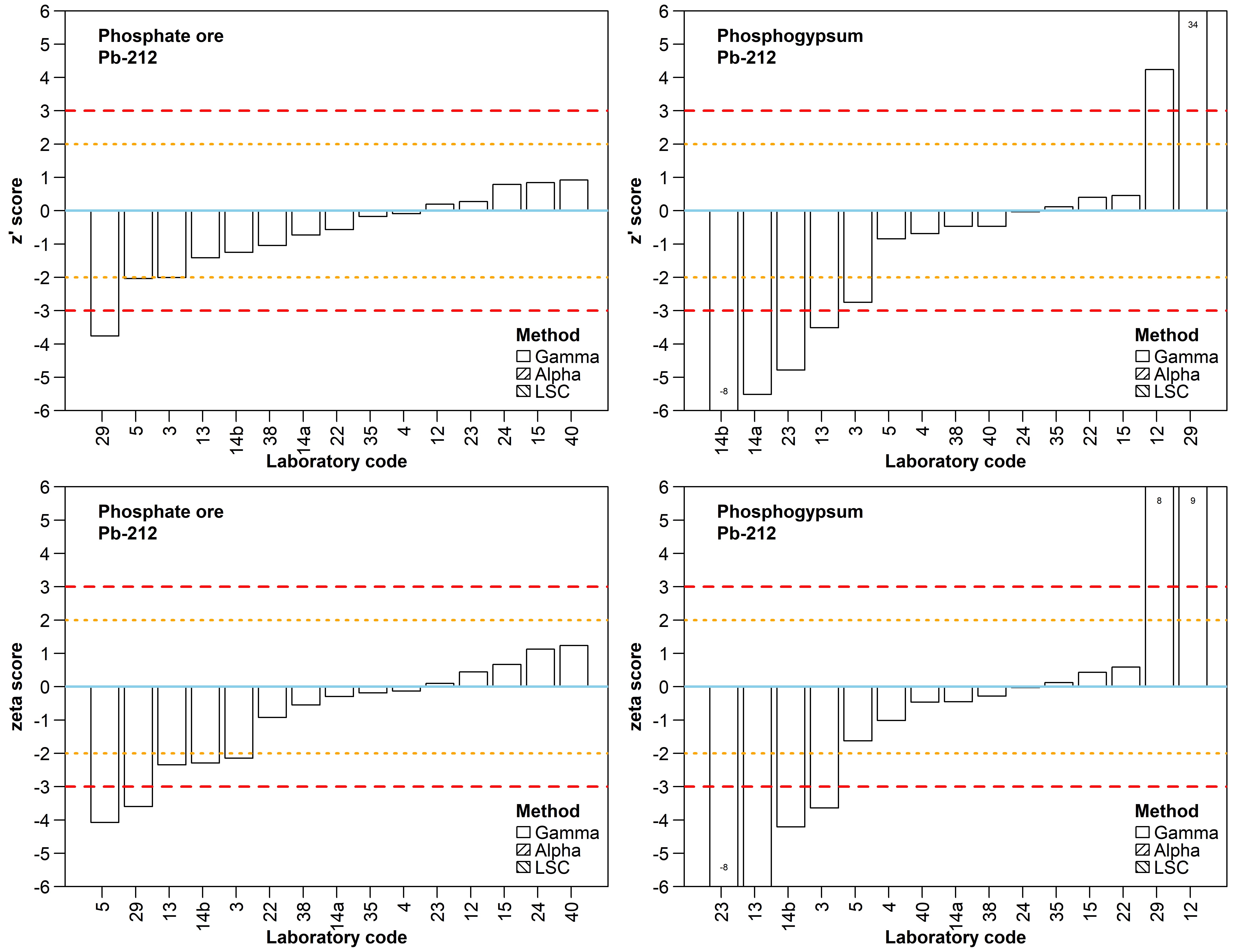

Supplement: Figure_S12_Pb-212_ncaf003 [file figure_s12_pb-212_ncaf003.jpeg]

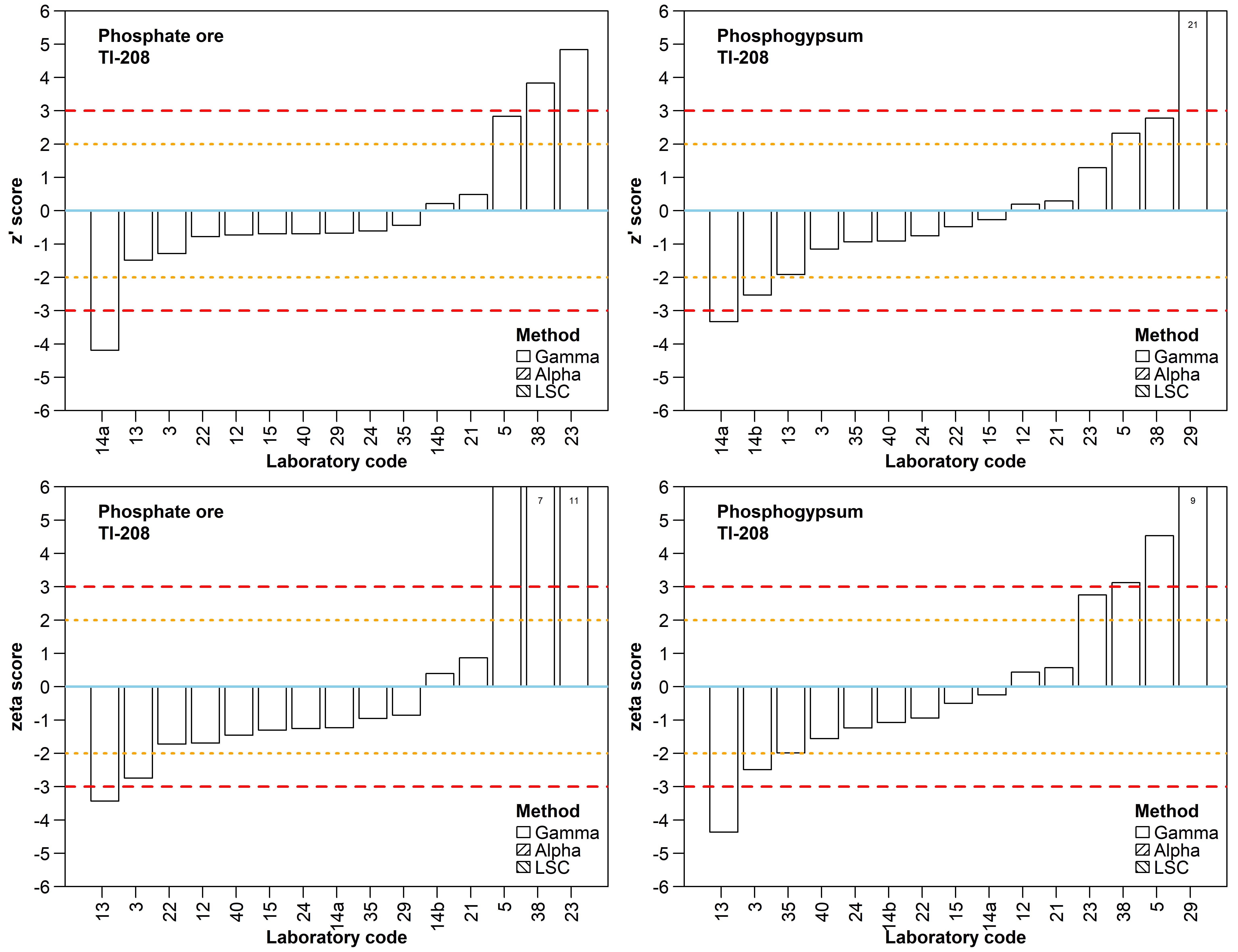

Supplement: Figure_S13_Tl-208_ncaf003 [file figure_s13_tl-208_ncaf003.jpeg]
