# Supplementary material for: Results of the joint IAEA/EEAE Intercomparison exercise on radioanalytical characterization of NORM samples in the European region
Source: Radiat Prot Dosimetry. 2025 Feb 20;201(3):223–46. doi: 10.1093/rpd/ncaf003 (PMC11884514; doi:10.1093/rpd/ncaf003)
Supplement: Supplementary_material_Appendix_1_Table_S1_ncaf003 [file supplementary_material_appendix_1_table_s1_ncaf003.pdf]

Supplementary material

Appendix 1 - List of the Official Participants in the Intercomparison Exercise

Table S1. List of the Official Participants in the Intercomparison Exercise.

**Laboratory Name**	**Member State**
Gamma Spectrometry Laboratory, Institute of Applied Nuclear Physics (IANP)	Albania
Environmental Monitoring Laboratory, Armenian Nuclear Power Plant (ANPP), Armenian Nuclear Regulatory Authority	Armenia
Faculty of Veterinary Medicine	Bosnia & Herzegovina
Radiation Protection Center	Bosnia & Herzegovina
National Center of Radiobiology and Radiation Protection	Bulgaria
Institute for Medical Research and Occupational Health (IMROH)	Croatia
Radioactivity Lab for Food and Environmental Samples, State General Laboratory	Cyprus
National Radiation Protection Institute	Czech Republic
Environmental Board, Climate and Radiation Department	Estonia
Mobile Laboratory, Georgian LEPL Agency of Nuclear and Radiation Safety (ANRS)	Georgia
Greek Atomic Energy Commission (EEAE)	Greece
Nuclear Engineering Department, NTUA	Greece
Institute of Nuclear & Radiological Sciences and Technology, Energy & Safety, NCSR Demokritos	Greece
teleDOS Laboratories SM PC - teleDOS Nuclear Tech	Greece
MERL, IO, Hellenic Centre for Marine Research (HCMR)	Greece
Department for Radiobiology and Radiohygiene, National Public Health Center	Hungary
Radiation Protection Centre, Ministry of Health	Lithuania
Radiation Protection and Noise Measurement Unit, Podgorica Center for Ecotoxicological Research	Montenegro
Institute of Public Health	North Macedonia
Institute of Nuclear Chemistry and Technology	Poland
Earth Sciences Department, University of Coimbra	Portugal
Radiation Protection, Environmental Protection and Civil Protection Laboratory, Institute for Nuclear Research	Romania
State Research Center – Burnasyan Federal Medical Biophysical Center of Federal Medical Biological Agency (SRC - FMBC)	Russian Federation
Department of Chemical Dynamics and Permanent Education, “Vinča” Institute of Nuclear Sciences	Serbia
National Institute of the Republic of Serbia, University of Belgrade, Department of Radiation and Environmental Protection, “Vinča” Institute of Nuclear Sciences	Serbia
Public Health Authority	Slovakia
Regional Authority of Public Health in Banská Bystrica	Slovakia
VUJE Trnava	Slovakia
Turkish Energy, Nuclear and Mineral Research Agency (TENMAK)	Türkiye
