# Supplementary material for: Results of the joint IAEA/EEAE Intercomparison exercise on radioanalytical characterization of NORM samples in the European region
Source: Radiat Prot Dosimetry. 2025 Feb 20;201(3):223–46. doi: 10.1093/rpd/ncaf003 (PMC11884514; doi:10.1093/rpd/ncaf003)
Supplement: Supplementary_material_Appendix_2_Table_S2_ncaf003 [file supplementary_material_appendix_2_table_s2_ncaf003.pdf]

Supplementary material

Appendix 2 - Validation of the data optimization procedure

To assess the optimization and the impact of excluding data from input in Algorithm A, there were a few control metrics in place. Firstly, the activity concentration ratios $^{214}Pb/^{226}Ra$ and $^{214}Bi/^{226}Ra$ are expected to be 1, irrespective of the ^{226}Ra determination method (e.g., gamma spectrometry, alpha spectrometry or liquid scintillation analysis), when a) NORM samples are appropriately sealed in radon-tight containers; b) the waiting time before gamma-spectrometric measurement is longer than approximately 30 days; c) the quality of the efficiency calibration of the spectrometer for the material under examination is high; d) all the required corrections are applied during spectrum analysis (e.g. true coincidence summing) and e) the nuclear data used are critically evaluated, up-to-date and coming from a reputed source such as LNHB. Secondly, the ratio $^{208}Tl/^{228}Th$ should be close to 0.3593, which is the decay branching ratio of ^{212}Bi to ^{208}Tl (LNHB, 2024). Thirdly, irrespective of the waiting time, the container radon-tightness and the ^{228}Ra determination method, the ratio $^{228}Ac/^{228}Ra$ should be close to 1. Finally, as far as the samples are natural materials, the activity concentration ratio $^{238}U/^{235}U$ should not be significantly different than 21.713 ± 0.043, considering abundance data for $N(^{238}U)/N(^{235}U)$ calculation from NIST (2021), and half-lives data for $T_{1/2}(^{235}U)/T_{1/2}(^{238}U)$ calculation from LNHB (2024). The closer the observed ratios are to the abovementioned ones, the better the optimization of the assigned values. The relative biases of the observed ratios are shown in Table S3, where it is evident that their absolute values were remarkably decreased after the optimization procedure. This confirms that the three reliability criteria adopted (see §2.4) operated well, as designed to function, and the optimized assigned values are expected to be closer to the true activity concentrations. Hence, the assigned values became more sensible after this data exclusion. It is also worth mentioning that if exclusion would not have taken place, the assigned values for almost all the radionuclides in both materials would be negatively biased by 6.1%, on average. This is a systematic or common bias that was successfully removed and should trigger warning or action signals to the laboratories that did not

fulfil the three reliability criteria (namely, laboratories #3, #5, #9, #13, #14a, #14b, #16, #19, #27, #29 and #34).

Table S2. Ratio bias prior and after data exclusion by sample type.

Assigned activities ratio	Ratio bias in phosphate ore sample (%)		Ratio bias in phosphogypsum sample (%)	
	Without data exclusion	After data exclusion	Without data exclusion	After data exclusion
$^{214}Pb/^{226}Ra$	-8.3	-3.2	-9.4	-3.9
$^{214}Bi/^{226}Ra$	-9.1	-2.7	-9.9	-2.8
$^{208}Tl/^{228}Th$	7.6	5.7	-28	-22
$^{228}Ac/^{228}Ra$	-4.9	-3.9	-11	-9.6
$^{238}U/^{235}U$	-14	-12	-43	-31
