# Supplementary material for: Results of the joint IAEA/EEAE Intercomparison exercise on radioanalytical characterization of NORM samples in the European region
Source: Radiat Prot Dosimetry. 2025 Feb 20;201(3):223–46. doi: 10.1093/rpd/ncaf003 (PMC11884514; doi:10.1093/rpd/ncaf003)
Supplement: Fig_ncaf003 [file fig_ncaf003.docx]

**Fig. S1.** Phosphate ore (left) and Phosphogypsum (right) ICE samples as distributed to the participants.

**Fig. S2.** Overall performance of the participants on the determination of the radionuclides of interest in the phosphate ore sample.

**Fig. S3.** Overall performance of the participants on the determination of the radionuclides of interest in the phosphogypsum sample.

**Fig. S4.** z´ and ζ scores for ^238^U by participant for the phosphate ore and phosphogypsum samples. Refer to the caption of Fig. 4 for a description of the line patterns used in z´ and ζ score plots.

**Fig. S5.** z´ and ζ scores for ^234^Th by participant for the phosphate ore and phosphogypsum samples. Refer to the caption of Fig. 4 for a description of the line patterns used in z’ and ζ score plots.

**Fig. S6.** z´ and ζ scores for ^214^Pb by participant for the phosphate ore and phosphogypsum samples. Refer to the caption of Fig. 4 for a description of the line patterns used in z´ and ζ score plots.

**Fig. S7.** z´ and ζ scores for ^214^Bi by participant for the phosphate ore and phosphogypsum samples. Refer to the caption of Fig. 4 for a description of the line patterns used in z´ and ζ score plots.

**Fig. S8.** z´ and ζ scores for ^210^Pb by participant for the phosphate ore and phosphogypsum samples. Refer to the caption of Fig. 4 for a description of the line patterns used in z´ and ζ score plots.

**Fig. S9.** z´ and ζ scores for ^228^Ra by participant for the phosphate ore and phosphogypsum samples. Refer to the caption of Fig. 4 for a description of the line patterns used in z´ and ζ score plots.

**Fig. S10.** z´ and ζ scores for ^228^Ac by participant for the phosphate ore and phosphogypsum samples. Refer to the caption of Fig. 4 for a description of the line patterns used in z´ and ζ score plots.

**Fig. S11.** z´ and ζ scores for ^228^Th by participant for the phosphate ore and phosphogypsum samples. Refer to the caption of Fig. 4 for a description of the line patterns used in z´ and ζ score plots.

**Fig. S12.** z´ and ζ scores for ^212^Pb by participant for the phosphate ore and phosphogypsum samples. Refer to the caption of Fig. 4 for a description of the line patterns used in z´ and ζ score plots.

**Fig. S13.** z´ and ζ scores for ^208^Tl by participant for the phosphate ore and phosphogypsum samples. Refer to the caption of Fig. 4 for a description of the line patterns used in z´ and ζ score plots.
